# New Chromones from *Bouvardia ternifolia* (Cav.) Schltdl with Anti-Inflammatory and Immunomodulatory Activity

**DOI:** 10.3390/plants12010001

**Published:** 2022-12-20

**Authors:** Yury Maritza Zapata Lopera, Enrique Jiménez-Ferrer, Maribel Herrera-Ruiz, Alejandro Zamilpa, Manasés González-Cortazar, Gabriela Rosas-Salgado, Mayra Alejandra Santillán-Urquiza, Gabriela Trejo-Tapia, Antonio Ruperto Jiménez-Aparicio

**Affiliations:** 1 Centro de Investigación Biomédica del Sur, Instituto Mexicano del Seguro Social, Xochitepec 62790, Morelos, Mexico; 2 Instituto Politécnico Nacional, Centro de Desarrollo de Productos Bióticos, Yautepec 62730, Morelos, Mexico; 3 Facultad de Medicina, Universidad Autónoma del Estado de Morelos, Cuernavaca 62209, Morelos, Mexico

**Keywords:** *Bouvardia ternifolia* root, rheumatoid arthritis, chromones

## Abstract

The extract, fractions, and compounds of the *Bouvardia ternifolia* root were evaluated as an antiarthritic using a complete Freund’s adjuvant (CFA) model in mice and NF-κB inhibition in RAW 264.7 macrophages. Four active compounds, including two new compounds, ternifoliol and ternifolial, were isolated by open column chromatography and identified by spectroscopic and spectrometric techniques, resulting in benzochromone-like structures with aromatic rings and hydroxyl groups, which could be responsible for the anti-inflammatory activity and inhibitory NF-κB. Changes in the joint cytokine profile monitored the antiarthritic effect. A decrement was observed in the local concentration of the following cytokines with different treatments: IL-17 by 64% and 70.3% with the aqueous extract (BtAq), ethyl acetate extract (BtAcOEt), and M3 fraction; interleukin-1 beta (IL-1β) by 10.2% and 15.7% with BtAq and the M4 fraction, respectively; IL-6 with M1, M2, M3, and M4 between 42% and 64%; necrosis factor-alpha (TNF-α) by 60.9% with M4. Conversely, the anti-inflammatory cytokine interleukin-10 (IL-10) increased between 94% and 99% with M1, M2, M3, and M4. Kidney IL-6 decreased with BtAq, M1, M2, M3, and M4 between 68.9% and 85.8%. TNF-α decreased with BtAcOEt, BtAq, M1, M2, and M4 between 34% and 80.2%. The NF-κB pathway was inhibited with BtAcOEt (90.1%), M1 (85%), M2 (93.5%), M3 (84.5%), M4 (90.3%), ternifoliol (75.6%), bouvardin (20.4%), and scopoletin (89%). We conclude that *B. ternifolia* modulated the inflammatory response at the joint and kidney levels and the NF-κB pathway.

## 1. Introduction

Rheumatoid arthritis (RA) is a chronic disease of inflammatory and autoimmune origin [1] that is linked to a genetic predisposition associated with the main class II histocompatibility complex [2]. In addition, it involves the differentiation between the Th1 or Th17 phenotypes by an antigen-presenting cell (APCs) and subsequent activation of B lymphocytes with consequent differentiation into plasma cells. On the other hand, macrophage activation triggers the inflammatory response of synovial cells. Activated B lymphocytes and macrophages are the main effectors of the expression and release of proinflammatory cytokines (IL-17, IL-6, IL-1β, INF-γ, and TNF-α). Activated synoviocytes stimulate osteoclasts and chondrocytes, which are responsible for the destruction of bone and cartilage [3]; furthermore, these same synoviocytes activate the secretion of cytokines, chemokines, matrix metalloproteinases, and cathepsins, leading to chronic inflammation and joint destruction [4]. Joint infiltration by activated macrophages plays a crucial role in the severity of RA and its symptoms. It is mediated by the production and release of TNF-α and IL-1β, which are involved in perpetuating the chronic inflammation associated with RA [5]. The previous immunological and inflammatory events are represented in the model of collagen-induced arthritis (CIA), in which animals are immunized with collagen, which is a specific component of the joint that is emulsified in Freund’s Complete Adjuvant. It is one of the most widely studied models of human rheumatoid arthritis. Specifically, NF-κB regulation of the genetic expression of the different molecules that mediate the inflammatory response is observed. NF-κB translocates to the nucleus when activated by IKK (cytoplasmic kinase), which removes IB, which complexes and inactivates NF-κB. When NF-κB enters the nucleus, it binds to specific DNA sequences, regulating the transcription of different genes involved in a wide variety of processes, such as proinflammatory cytokines and chemokines, adhesion to iNOX, COX-2, and other molecules [6]. Therefore, natural products that inhibit NF-κB have been described as anti-inflammatories or immunomodulators [7]. *Bouvardia ternifolia* (Cav.) Schldtl (Rubiaceae), commonly known as “trumpet,” is distributed in Mexico and the United States.

In traditional medicine, the aerial parts of the plant (leaves, stems, and flowers) are used to treat genital ulcers, dysentery and rabies, cold sweat pains, tumors, fever, and joint pain, and as a fortifier. It is also a sedative, analgesic, and antispasmodic and are used for treating snake bites, bee stings, scorpion stings, and spider bites. In addition, the ancient indigenous people used root powder to heal sores and stop bleeding (hemostatic) [8].

The pharmacological activities of *B. ternifolia* are cytotoxic, antitumor, and antivenom; in this last activity, the root of the plant is used [9]; the competitive inhibition activity of the enzyme acetylcholinesterase is used as a treatment for Alzheimer’s disease [10]. The aerial parts of the plant show antioxidant and anti-inflammatory effects [11]. All the pharmacological properties previously exposed are due to the presence of secondary metabolites such as ursolic acid, oleanolic acid, chlorogenic acid, 3-O-quercetin rhamnopyranoside, 3-O-quercetin glucopyranoside, rutin, and scopoletin, which have anti-inflammatory properties [10]. This work aims to explore the immunomodulatory effect of *Bouvardia ternifolia* (Cav.) Schltdl in an experimental arthritis model by measuring the cytokine profile and the anti-inflammatory effect on the activation of NF-κB and, on the other hand, to broaden the knowledge about the chemical constitution of the species, defining the chromatographic profile and the structural elucidation of the isolated active components, by spectroscopic methods.

## 2. Results

### 2.1. Chemical Analysis

**Figure 1:**
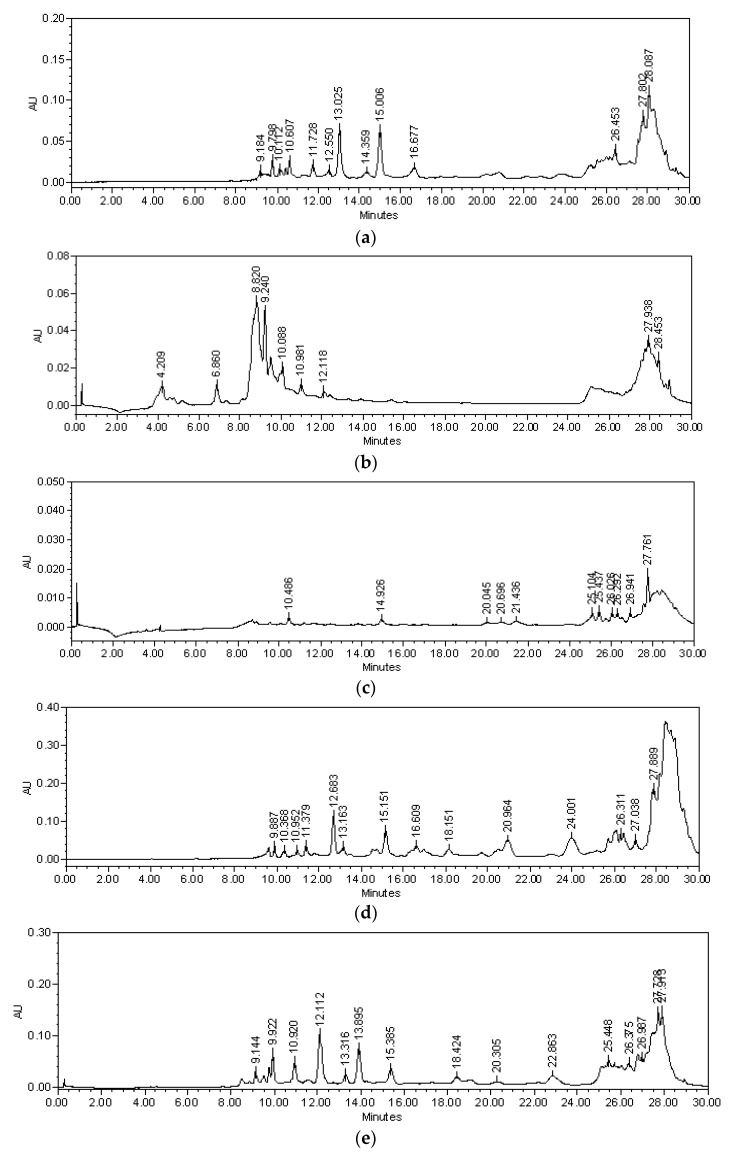

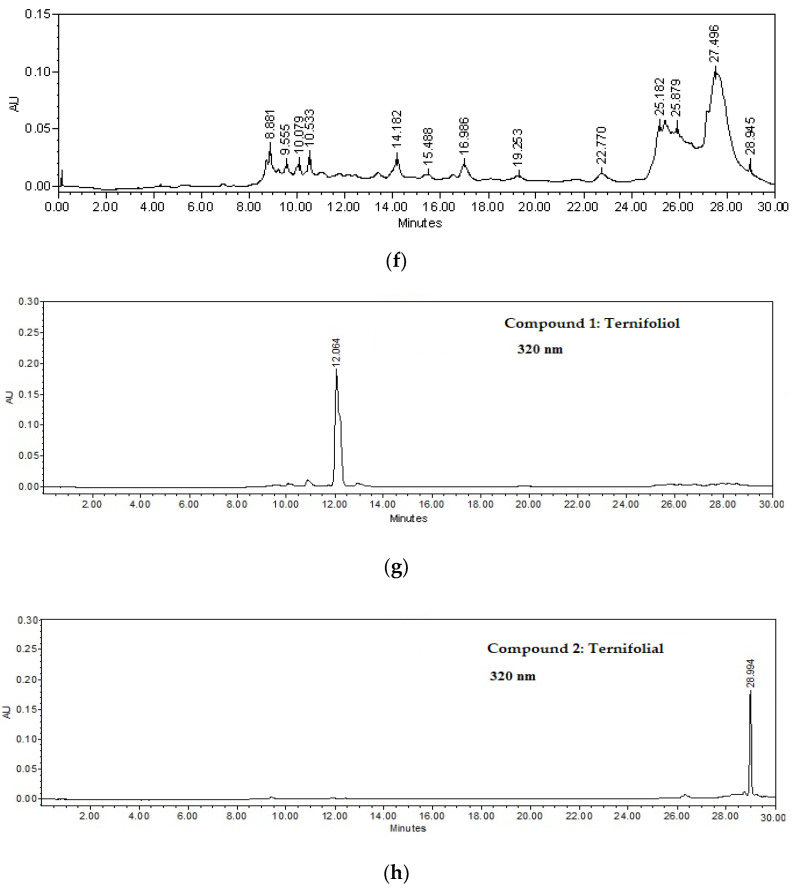

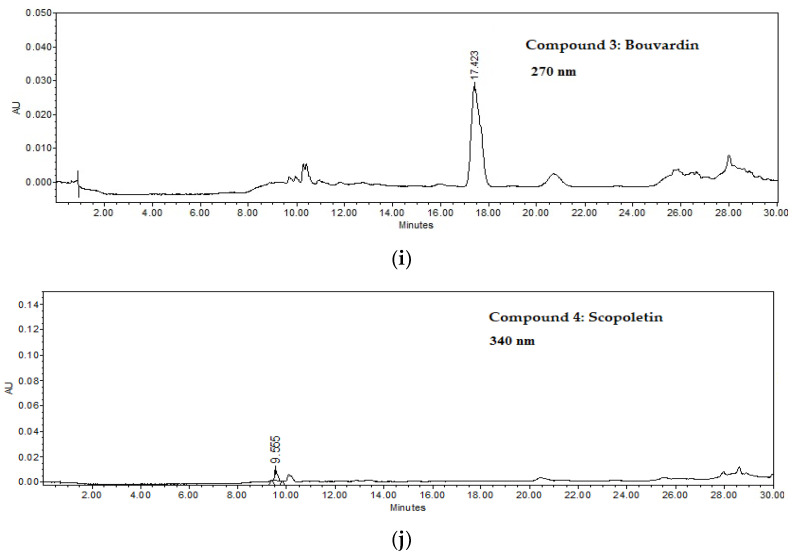
Chromatographic profiles obtained by HPLC of the extracts, fractions, and compounds obtained from the root of *Bouvardia ternifolia*: (**a**) Bt-AcOEt (270 nm); (**b**) Bt-Aq (270); (**c**) M1(320); (**d**) M2 (270); (**e**) M3 (270); (**f**) M4 (270); (**g**) ternifoliol (**1**) (12.064 min; (**h**) ternifolial (2) 28.994 min; (**i**) bouvardin (**3**) 17.423 min; (**j**) scopoletin (**4**) (9.55 min).

**Figure 2:**
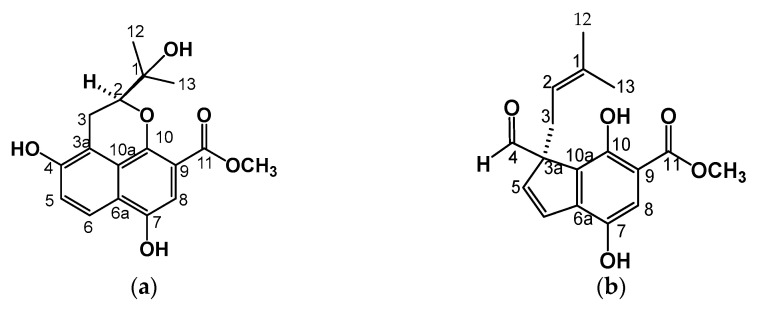

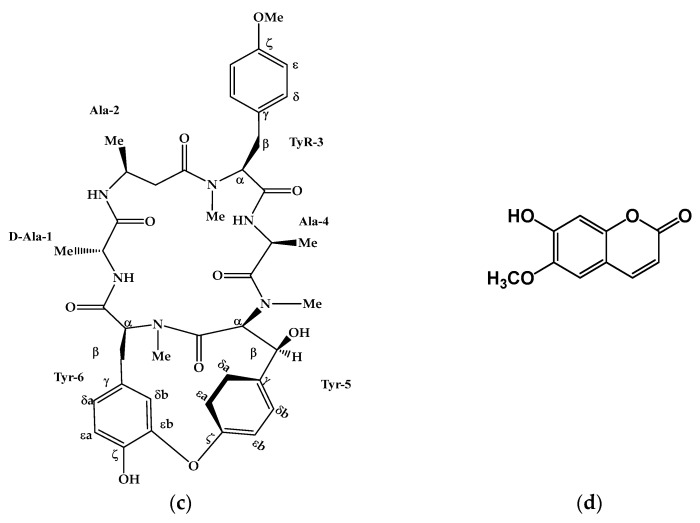
Compounds isolated and characterized from the roots of *Bouvardia ternifolia*: (**a**) ternifoliol (**1**); (**b**) ternifolial (**2**); (**c**) bouvardin (**3**); (**d**) scopoletin (**4**).

A bioassay-guided chemical investigation led to the identification of four bioactive compounds from the roots of *Bouvardia ternifolia* (Figure 1 and Figure 2).

In the Bt-AcOEt extract (Figure 1a), ternifoliol (Rt = 12.064 min) could be identified as the majority compound, followed by ternifolial (Rt = 28.994 min). Bouvardin (Rt = 17.423 min) and scopoletin (Rt = 9.555 min) were identified in a smaller proportion. The Bt-Aq extract (Figure 1b) presents lower concentrations of the two chromones without detecting bouvardin; however, scopoletin was present in higher concentrations, making it possible to define it as the majority compound. In the M1 fraction (Figure 1c), ternifoliol and ternifolial were also identified, although in a much smaller proportion than Bt-AcOEt. Bouvardin and scopoletin were not detected. Finally, it is worth mentioning that a group of unidentified medium polarity compounds is presented. The M2 fraction (Figure 1d) is one of the fractions used in which the highest concentration of ternifoliol is comparatively identified, with a lower amount of ternifolial and an amount of bouvardin comparable to what was detected in Bt-AcOEt. The M3 fraction (Figure 1e) presented a lower concentration of ternifoliol and a higher concentration of ternifolial compared to M2. Regarding bouvardin, it has a very similar concentration to M2 and Bt-AcOEt. What is remarkable is that one of the highest concentrations of scopoletin is observed. The M4 fraction (Figure 1f) occupies the second place in the composition of ternifoliol after M2.

Regarding ternifolial, this fraction presents the highest concentration of this compound. Similarly, both bouvardin and scopoletin have the highest concentrations compared to the other fractions.

### 2.2. Isolation of Compounds **1**–**4**

#### 2.2.1. Ternifoliol (**1**)

This compound is a powder that is soluble in CH_3_OH. TLC showed a blue, fluorescent band when observed under UV light (λ = 365 nm). HPLC showed a peak at 12.064 min at 320 nm and absorption spectrum λmax = 206, 229, and 345 mn, characteristic of a coumarin-type chromone (Figure 1g). Compound **1** displayed a quasimolecular positive ion at *m/z* 341 [M + Na]^+^ in ESI MS (Supplementary Figure S8), with molecular formula C_17_H_18_O_6_. The IR spectrum (Supplementary Figure S30) exhibited a broad absorption band of hydroxyl (3393.28 cm^−1^), C-H stretch aromatics (2923.22 cm^−1^), C-H stretch alkyl (2851.26 cm^−1^), out-of-plane, or “oop”, bands (900–675 cm^−1^), and “oop” C-H bending (740, 45 cm ^−1^). Specific rotation of **1** [α]^20^_D_ = +8°.

**Figure 3:**
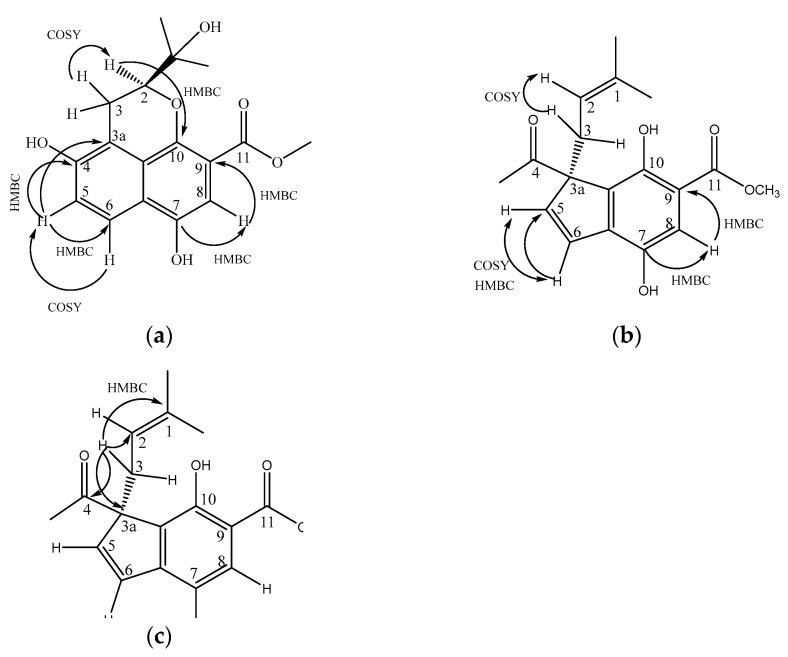
Correlations of ternifoliol and ternifolial ^1^H, ^13^C (HSQC) and ^1^H, ^13^C (HMBC) ^1^H (COSY) NMR (CDCl_3_, 600MHz): (**a**) HMBC and COSY ternifoliol; (**b**) COSY ternifolial; (**c**) HMBC ternifolial.

The ^1^H-NMR spectrum analysis of **1** (Table 1) showed two aromatic ring systems: an AB system in δ 7.86 (dd, J = 0.7, 9.1) and δ 7.13 (d, J = 9.1), which corresponded in the HSQC experiment to C-5 carbon (δ 120.6) and C-6 carbon (δ 123.9), respectively (Supplementary Figure S6). The A system had a signal at δ 6.90 (s, H-8), which, according to HSQC, corresponded to C-8 carbon (δ 105.7). In the HMBC (Supplementary Figure S7) H-5 (d, J = 7.86) experiment, this correlated with the signals on C-6 carbon (δ 123.9), C-4 carbon (δ 147.9), and C-3a carbon (δ 115.9). The COSY experiment corroborated these couplings. (Figure 3).

The H-8 (δ 6.90) correlated in HMBC with the signal at the C-7 carbon (δ 149.4) of hydroxyl and with the C-9 carbon (δ 169.2), which was an ester carbonyl. This analysis determined that the union of these rings is the base structure of naphthalene.

A third ring is formed with a signal at δ 3.81 (s, br) that was assigned to H-2 and bound to heterocyclic oxygen, which was confirmed by the correlation to two and three bonds of H-2 (δ 3.81, s, br) with the C-10 carbon (δ 152.6) and which, in turn, correlates with the signal at δ 72.9, which is the C-1 carbon-based alcohol.

In the proton, there is the presence of methyl signals at the C-12 (δ 25.9) and C-13 (δ26.4)) carbons that are linked with the C-1 carbon. In the COSY experiment (Supplementary Figure S5), H-2 was coupled with H-3, which showed signals at δ 3.20 (dd, J = 3.6, 13.5) and δ 2.72 (dd, J = 3.6, 13.5) assigned to methylene, and interacted in HMBC with signals C-3a (δ 115.9) and C-4 (δ 147.9), which were bound to an OH. According to the 1 and 2D NMR (Figures S2–S7) data and comparison with a similar molecule, the skeleton formed by two rings and a heterocycle corresponded to 4,7-Dihydroxy-2- (1-hydroxy-1-methyl-ethyl) -2, 3-dihydro-benzo [de] chromene-9-carboxylic acid methyl ester, which is a newly described Benzo [de] chromene that we named ternifoliol (**1**) and is firstly reported in this work (Figure 2a). This compound was purified from the BM4 fraction from the M3 fraction.

#### 2.2.2. Ternifolial (**2**)

Ternifolial is a deep-yellow precipitate that was soluble in dichloromethane; TLC showed a blue, fluorescent band when observed under UV light λ = 365 nm; HPLC showed a peak at 28.994 at 320 nm and absorption spectrum λmax = 201, 319, and 405 mn (Figure 1h), characteristic of a coumarin-type chromone. In addition, compound **2** displayed a quasimolecular positive ion at m/z 304 [M + 2H]^+^ in ESI-MS (Supplementary Figure S17) with molecular formula C17H18O5. The IR spectrum (Supplementary Figure S31) exhibited a broad absorption band of hydroxyl (3354.26 cm^−1^), C-H stretch aromatics (2922, 85 cm^−1^), and C-H stretch alkyl (2852.13 cm^−1^), out-of-plane or “oop” bands (900–675 cm−^1^), and in-plane C-H bending (1020.34 cm^−1^) functional groups. The specific rotation of **2** is shown by [α]^20^_D_ = +15°.

In the ^1^H-NMR and ^13^C-NMR spectrum analysis, **2** (Table 1) showed a double bond at δ 7.88 (d, J = 9.9) coupled with the signal at δ 6.21 (d, J = 10.27), assigned H-5 and H-6, respectively, in a cyclopentane. In HMBC, H-5 and H-6 correlated with the carbon signals at δ 56.7, assigned to carbon C3a and methylene at 37.2 (δ3.41, dd, J = 7.3, 13.9) and δ 2.74 (dd, J = 7.3, 13.9), corresponding to H-3a and H-3b, respectively, and, finally, with the signal of an aldehyde carbonyl at δ 204.9.

H-3 was part of an isoprene unit that, according to the COSY experiment (Figure 3b), was coupled to a vinyl signal at δ 4.56 (dd, J = 7.3, 8.8) and assigned to H-2, which is coupled at a long distance to C-1, C-12, and C-13 at δ 133.6, 25.6, and 17.7, respectively. In ^1^H NMR, the signal at δ 7.28 (s, br) corresponded to a pentasubstituted aromatic ring. The HMBC experiment allowed us to determine the correlations with the signals δ 127.57, 154.1, 112.2, 144.7, and 127.53 assigned to C-6, 7, 9, 10, and 10, respectively (Figure 3c). Additionally, a methoxy group was attached to C-11 (δ 170.6) of an ester. According to the 1 and 2D NMR data (Supplementary Figure S10–S16), this compound could be identified as 3-Formyl-4,7-dihydroxy-3- (3-methyl-but-2-enyl) -3H-indene-5-carboxylic acid methyl ester (Figure 2b).

The second compound was identified and reported for the first time for this plant and was called ternifolial (2). This compound also was purified from M3 fraction and BM5 subfraction. Both compounds, ternifolial and ternifoliol, are chromones.

#### 2.2.3. Bouvardin (**3**)

This molecule is a yellow powder that was soluble in CH_3_OH. HPLC showed a peak at 17.423 min at 270 nm (Figure 1i) and a spectrum with λmax of 204 and 275nm; compound **3** displayed a quasimolecular positive ion at m/z 773.41 [M + H]^+^ in ESI-MS (Supplementary Figure S25), with molecular formula C₄₀H₄₈N₆O₁₀, which coincides with bouvardin PM 772.86 g/mol [12]. It presents 38 signals. Three signals at 30, 33, and 40 ppm indicated three N-methyl-L-tyrosines at 55 ppm for tyrosine-3-O-methyl, an AB system at positions 115 ppm and 130 ppm, and the presence of a hydroxyl group attached to tyrosine 5 at 160 ppm, and the six carbonyl groups linked to peptides (171, 172, 173, and 174 ppm) together suggest the structure of bouvardin (Figure 2c) as well as the presence of the hydroxyl group in tyrosine -5-β (Figures S19–S24). This compound was also purified from BM6, a subfraction from M3.

#### 2.2.4. Scopoletin (**4**)

This metabolite is in the M1 fraction with a peak at 9.555 min at 340 nm (Figure 1j) and a spectrum with λmax = 229, 297, and 345 nm. Comparison with standards in both TLC and HPLC identified it as scopoletin (Figure 2d). In addition, ^1^H-NMR and ^13^C-NMR also identified it (Figures S27–S29).

This compound also was purified from the M2 fraction. So, then, in fractions, M2 and M3 are the most abundant components of Bt-AcOEt. Additionally, they are the chromones of a recent discovery in the plant.

### 2.3. Anti-Inflammatory Activity of B. ternifolia on Joints Edema Induced with Freund’s Adjuvant

**Figure 4:**
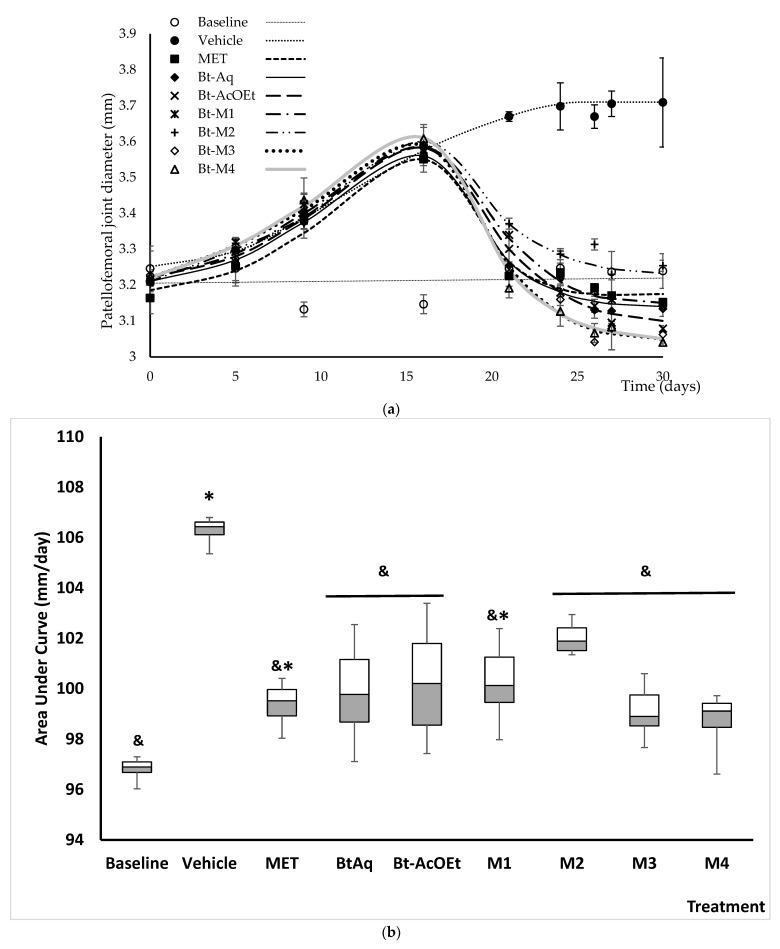
Temporal course of inflammation in the patellofemoral joint of mice with experimental arthritis (**a**) under the different treatments obtained from the *B. ternifolia* extract. The area under the curve (AUC) of the temporal course of the patellofemoral joint inflammation evolution and the effect of different experimental treatments obtained from *B. ternifolia* extract applied to mice with experimental arthritis (**b**). It is compared against the negative control groups or treatment with Vehicle (^&^) and basal control (*). The aqueous extract BtAq and the ethyl acetate extract, BtAcOEt, were derived from the bipartition of the complete extract. Fractions M1, M2, M3, and M4 were obtained from BtAcOEt. ANOVA with post hoc Tukey tests with * *p* < 0.05 compared with baseline group, ^&^ *p* < 0.05 compared with Veh group (*n* = 7 ± SD).

Figure 4a shows the temporal course of the inflammatory response of the patellofemoral joint of mice with experimental arthritis. The basal group showed an articular radius that remained relatively constant during the month of evolution and ranged from 3.15 to 3.25 mm. The group with joint damage (Veh) showed an upward trend in the diameter of the joint, reaching a maximum value of 3.7 mm, representing a 15% swelling. All the treatments tested, including methotrexate (MTX), decreased the articular edema induced with Freund’s adjuvant. The treatments showed a trend in their effectiveness, with the most effective being M3 and M4, which were obtained from Bt-AcOEt, the next most effective, followed by treatments M1, Bt-Aq, and MTX. In the end, we found M2. However, that significantly reduced the patellofemoral inflammation observed in the damage group (Vehicle). It can be seen more clearly in Figure 4b, which shows the area under the curve (AUC).

Figure 4b shows the AUC from the evolution of the inflammation of the patellofemoral joint over time. The method used for AUC determination was the sum of trapezoids. It was established that the treatment for the vehicle or damage group showed an increase of 10.4% in this parameter. MTX is in a class of medications called disease-modifying drugs of the first choice in RA. In this work, such treatment was able to significantly reduce joint diameter compared to the damage group, although the diameter of the baseline control group was not reached. Furthermore, all experimental treatments decreased significantly compared to the Veh group (^&^*p* < 0.05).

### 2.4. B. ternifolia on the Cytokine’s Levels in Kidney and Joints of Mice RA-Induced with Freund’s Adjuvant

**Figure 5:**
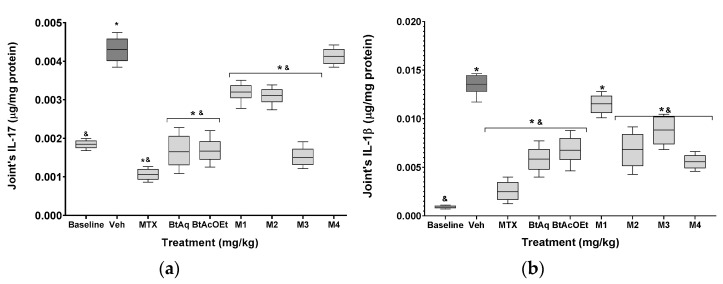

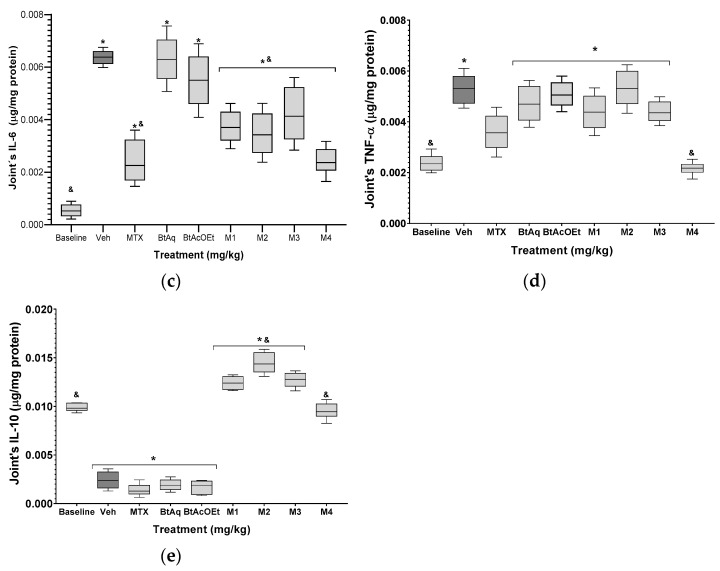
Effect of different experimental treatments obtained from *B. ternifolia* extracts on the inflammatory response (**a**) IL-17, (**b**) IL-1β, (**c**) IL-6, (**d**) TNF-α and (**e**) IL-10, of the patellofemoral joint of mice with experimental arthritis, compared to negative control Vehicle (^&^) and Basal control (*) groups. The aqueous extract BtAq and the ethyl acetate extract, BtAcOEt, were derived from the bipartition of the complete extract. Fractions M1, M2, M3, and M4 were obtained from BtAcOEt. ANOVA with post hoc Tukey tests with * *p* < 0.05 compared with baseline group, ^&^ *p* < 0.05 compared with Veh group (n = 7 ± SD).

The cytokine concentration in joints from mice with experimental RA is shown in Figure 5; the damaged group (Veh) presented a significant increase in IL-17, IL-1β, IL-6, and TNF-α, with 2-, 13-, 12-, and 3-fold increases, respectively, in comparison with the basal group (* p < 0.05). Treatment with MTX significantly counteracts the effect of Freund’s adjuvant on the proinflammatory cytokines by decreasing its value compared to Veh (* *p* < 0.05), although it does not modify IL-10 (*p* > 0.05). The experimental treatments of the BtAq and BtAcOEt of *B. ternifolia* root significantly reduced the level of IL-17 and IL-1β, but not concentrations of the other proinflammatory cytokines, and did not significantly change the levels of the anti-inflammatory cytokine IL-10. The treatments with the M1, M2, M3, and M4 fractions obtained from the BtAcOEt were more effective in reducing the proinflammatory environment compared to BtAq and BtAcOEt. IL-17 decreased significantly with M1, M2, and M3, although the latter’s effect was much more significant. IL-1β significantly decreased with M2, M3, and M4. The differences in IL-6 among treatments were as follows: M4 > M2 > M1 > M3. TNF-α decreased significantly only with M4. All four fractions increased IL-10 concentration, with the following trend: M2 > M3 > M1 > M4.

**Figure 6:**
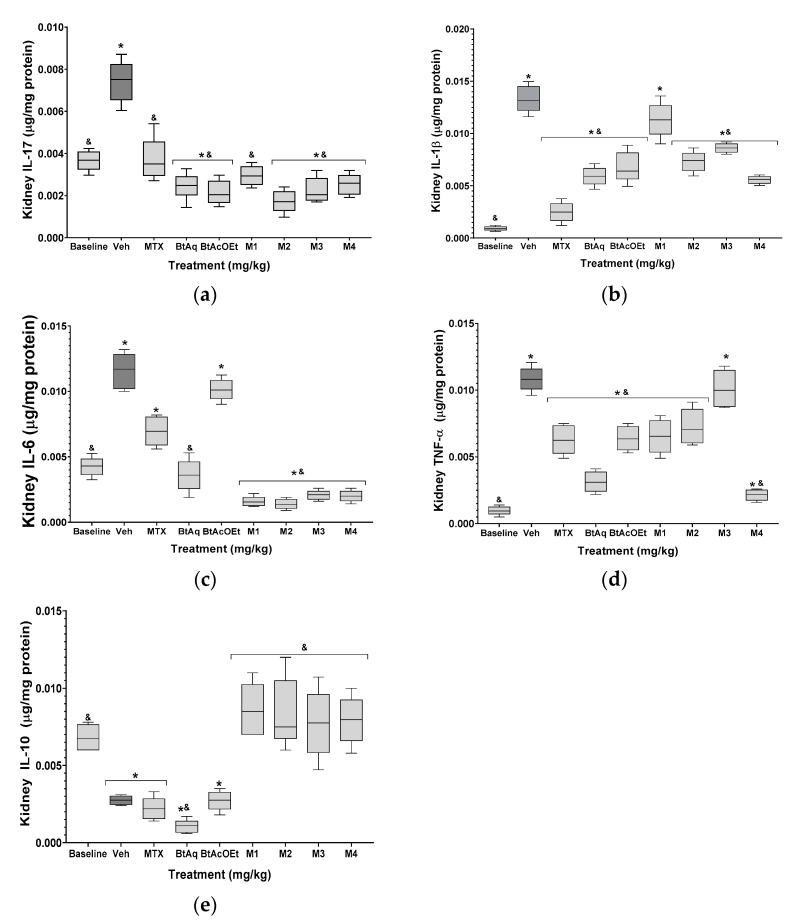
Effect of different treatments obtained from the *B. ternifolia* root extract on the inflammatory response (**a**) IL-17, (**b**) IL-1β, (**c**) IL-6, (**d**) TNF-α and (**e**) IL-10 of the kidneys of mice with experimental arthritis. The aqueous extract BtAq and the ethyl acetate extract BtAcOEt were derived from the bipartition of the complete extract. Fractions M1, M2, M3, and M4 were obtained from BtAcOEt. ANOVA with post hoc Tukey test, with * *p* < 0.05 compared with baseline group, ^&^ *p* < 0.05 compared with Veh group (*n* = 7 ± SD).

Figure 6 shows the response of the kidneys of mice with experimental arthritis. Significant increases in the concentration of IL-17, IL-1β, IL-6, and TNF-α (2-, 14-, 2.7-, and 11-fold increases), and IL-10 showed a significant 2.5-fold decrease. Like cytokine levels from the patellofemoral joint, methotrexate decreased the kidney concentrations of the four proinflammatory cytokines in the patellofemoral joint. However, the level of IL-10 was not recovered in the healthy group. The experimental treatments (BtAq and BtAcOEt) improved the proinflammatory environment, as evidenced by a significant decrease in the renal levels of IL-17, IL-1β, IL-6, and TNF-α, in contrast to the patellofemoral joint. However, BtAcOEt was the exception to this trend, showing a significant increase in renal IL-6. Regarding IL-10, it did not recover the renal concentration of the group of healthy mice with the BtAq and BtAcOEt treatment since the renal level of IL-10 was not different from that of the damaged control group. Treatments with the M1, M2, M3, and M4 fractions obtained from BtAcOEt effectively reduced the proinflammatory environment associated with RA. The renal concentration of IL-17 had the following behavior: M2 > M3 > M4 > M1. IL-1β showed the following trend M4 > M2 > M3; with M1, there was no significant difference with the damage group. The order of IL-6 was: M2 > M1 > M4 > M3, and for TNF-α, the trend was: M4 > M1 > M2. Treatment with M3 did not significantly decrease the renal concentration of TNF-α compared to the damaged control group. All the treatments increased renal concentration of IL-10 in the following order: M1 > M2 > M4 > M3.

### 2.5. Effect of Fractions and Compounds from B. ternifolia on the Expression of NF-κB In Vitro

RA leads to inflammation of the joints’ synovial membrane, with progressive destruction of the articular cartilage and underlying bone. These structural alterations are accompanied by pain and functional limitation. The inflammatory process is mainly mediated by the production of cytokines, growth factors, chemokines, and synovial cells. This study was conducted to evaluate different anti-inflammatory and immunomodulatory treatments of *B. ternifolia* in an experimental RA assay induced with Freund’s adjuvant and to determine whether the isolated compounds inhibited the expression of NF-κB as part of the mode of action [13].

**Figure 7:**
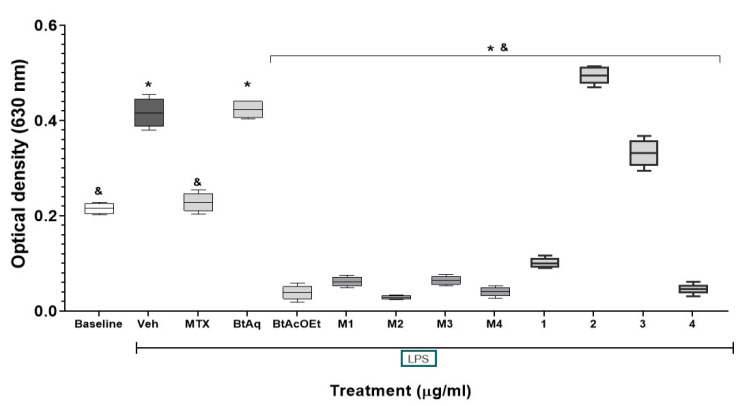
Inhibitory effect of the extracts, fractions, and compounds of *B. ternifolia* root on the expression of NF-κB in Raw-Blue 264.7 macrophages. We applied an LPS stimulus in all treatments except for the basal expression group. The aqueous extract BtAq and the ethyl acetate extract BtAcOEt were derived from the bipartition of the complete extract. Fractions M1, M2, M3, and M4 were obtained from BtAcOEt. Ternifoliol (**1**), ternifolial, (**2**) bouvardin (**3**), and scopoletin (**4**) were isolated from previous fractions. ANOVA with post hoc Tukey test with ^&^ *p* < 0.05 compared with Veh group (*n* = 6 ± SD). Comparison against negative control damage group (*).

The chemical profile of *B. ternifolia* was characterized by secondary metabolites, including terpenes, phenolic compounds, and cyclopeptides. This paper describes two new compounds: ternifoliol and ternifolial. These were isolated from subfractions BM4 and BM6; ternifoliol showed inhibitory activity of NF-κB (Figure 7).

## 3. Discussion

Currently, the factors that cause the appearance of the symptoms of RA are unknown [14]. However, the succession of immunological events in the sinus delimited by the synovial membrane, a compartment occupied by the synovial fluid, is clear. First, activated synovial macrophages release cytokines such as TNF-α, IL-1β, and IL-6, which have a proinflammatory function [14,15]; at the same time, the stimulation of fibroblasts-like synoviocytes (FLS) is carried out, stimulating the activity of osteoclasts, which leads to bone erosion; this is because activated FLSs are capable of producing matrix metalloproteinases [3,14] and, thereby, direct the degradation of cartilage. On the other hand, activated FLS stimulates the expression of receptor activator of nuclear factor-κB ligand (RANKL), which leads to T cells binding to proteins on the surface of osteoclasts, causing bone erosion by increasing activity thereof [14]. Similarly, reactive oxygen species (ROS) released during the described acute inflammatory process can activate NF-κB, which activates type 1 helper T cells (Th1) [15], thereby causing the production and release of proinflammatory cytokines TNF-α, IL-1β, and IL-6 [16]. This environment generated by the described cytokines promotes the maturation of resting monocytes into mature dendritic cells, leading to the presentation of autoantigens to autoreactive T lymphocytes, directing the inflammatory response to target tissues and promoting inflammation and erosion of the joints. At the same time, the oxidative stress caused by the increased tissue levels of ROS leads to the citrullination of arginine peptide residues, which establishes systemic inflammation [14,17], which is mediated by anticitrullinated protein antibodies (ACPAs), which also triggers the immune response by binding to cell receptors and activating the complement system, enhancing joint inflammation and bone erosion [14,18]. As mentioned, bone erosion and cartilage degradation resulting from stimulating RANKL expression, with associated IL-17 production and release, are seen as the most apparent deleterious condition that causes RA [18,19].

As already stated, RA is an autoimmune inflammatory disease that primarily affects small and large joints. However, a series of extra-articular structures are also affected, defined as extra-articular manifestations that correlate with high morbidity and mortality rates [20]. Extra-articular manifestations are generally associated with the production and release of proinflammatory cytokines. One of the most severe extra-articular manifestations is vasculitis, which forms part of the most widespread manifestations, such as skin conditions, gastrointestinal complications, heart diseases, and pulmonary and renal manifestations. Regarding the kidney, the specific function of glomerular filtration is the condition that shows the most significant impact due to tissue damage derived from the plasmatic circulation of proinflammatory cytokines and other cellular components of the inflammatory response [21]. The model of collagen-induced arthritis (CIA) reproduces many of the characteristic events of RApathophysiology, which is why it is widely used in evaluating new treatments, such as synthetic ones and those derived from medicinal species. For example, *B. ternifolia* has been used in traditional Mexican medicine against inflammation-related problems. That activity is shown for extracts and fractions of this plant using ear edema with a phorbol ester in mice [9]. This paper shows this plant’s antiarthritic and immunomodulator effect on extracts, fractions, and compounds. The chemical study allowed an approach that led to the isolation of structurally and pharmacologically novel compounds. As observed in the results of the CIA model, a significant decrease in joint edema of the patellofemoral joint of mice with experimental arthritis is shown over time. It is comparable to the response to MTX, where M3 and M4 were the most effective treatments. The M3 fraction presented a great diversity in its composition, highlighting the presence of scopoletin, ternifoliol, and ternifolial. Similarly, M4 showed great complexity in its composition, although the concentrations of the active ingredients were lower than M3, where we identified scopoletin and, prominently, ternifolial. In the sequence of treatment effectiveness, we found BtAcOEt and M1 (Figure 1a,b, respectively), in which the presence of ternifolial stands out. Ternifolial belongs to a group of chromone compounds whose activities have been described as inhibiting proinflammatory cytokines TNF-α and IL-6, inhibiting NF-κB transcriptional activity and decreasing iNOS expression [22]. In the Bt-AcOEt extract, ternifoliol could be identified as the main compound, followed by ternifolial, as well as bouvardin and scopoletin, although to a lesser extent. These data contrast with the Bt-Aq extract (Figure 1b), which presents lower concentrations of the two chromones without the presence of bouvardin; however, scopoletin was present in higher concentrations and can be defined as the main compound.

Scopoletin has been described in various anti-inflammatory and antioxidant activities [23,24], especially in identifying the modulation of NF-κB expression, mediated by Nrf2 [25], which allows us to suppose that both the extracts and fractions of *B. ternifolia* have a fully justified anti-inflammatory effect. In the joints, it is observed that the pathophysiological process of RA that developed secondary to the administration of CFA caused a significant increase in the production of TNF-α, IL-6, and IL-1β [3]. Allthe tested treatments derived from *B. ternifolia*, M4 could prevent the production of these proinflammatory cytokines, which may be related to ternifolial, which, as already mentioned, has an anti-inflammatory [23,24] and immunomodulatory effect [25]. Similarly, RA generates a significant increase in IL-17, which is associated with CD4+ T cells that promote inflammation, bone erosion, and cartilage degradation by the stimulating expression of NF-κB-specific activator receptor ligand (RANKL) and produce IL-17, which stimulates synovial macrophages and FLS [26,27] and to plasma cells that also promote inflammation through the production of cytokines and autoantibodies [14,28]; treatments based on extracts and fractions of *B. ternifolia* decreased the level of IL-17, particularly M3. The presence of ternifolial, ternifoliol, and scopoletin could be responsible for this anti-IL-17 effect [23,24,25]. Regarding the response mediated by IL-10, it should be considered that RAresults from subverted immunological tolerance [29]. As mentioned, the sequence of critical events is the expansion of Th1 and Th17 cell lines and TNF-α production cells.

In contrast, there is a decrease in regulatory T cells (Treg), which limits the activity of proinflammatory T cells. Similarly, autoreactive B cells consolidate the inflammatory response by synthesizing autoantibodies and proinflammatory cytokines, which are counteracted by regulatory B cells (Breg) [30,31]. The absence of Breg cells causes the development of severe RA in a murine model [32]. From this, the hypothesis has arisen that increasing the number of functional Breg cells in animal models of RA and patients suffering from RA could generate a regulatory response on the inflammatory response [30]. In patients suffering from RA, it has been shown that mature Breg cells can decrease the levels of Th1 and Th17 TNF-α + cells and increase Treg and Tr1 cells through the production of IL-10 [33,34,35].

Regarding IL-10 (Figure 5e), a significant increase is shown in the treatments with M1, M2, M3 and M4 compared to the treatments with Veh, MTX, BtAq and BtAcOEt. It seems to indicate that control of the inflammatory response, evaluated by the level of proinflammatory cytokines (TNF-α, IL-1β, and IL-6), occurred in a differentiated manner. Since, in the first case, proinflammatory cytokines decreased independently of IL-10, and with fractions from M1 to M4, it was dependent on the production of IL-10.

Renal tissue production of cytokines was evaluated to evaluate extra-articular damage produced by CFA. The inflammatory response of the kidney showed a different behavior than the inflammatory process of the patellofemoral joint. In TNF-α, treatment with BtAq shows greater efficacy in the kidney compared to the joint. Regarding IL-1β, a similar response was presented in the joint and the kidney. With IL-6, the BtAq and M1 to M4 treatments were more effective because they decreased the tissue levels of IL-6. With IL-17, the treatments presented were very differentiated; the treatments with M1 to M4 presented the most significant effect in the kidney. The results with *B. ternifolia* in the culture cells Raw-Blue indicate that the treatments reduced NF-κB significantly, notably, Bt-AcOEt, M1, M2, M3, M4, ternifoliol, and bouvardin. These data are relevant in the evolution of RA whit these treatments since dysregulated activation of NF-κB also contributes to the aberrant survival of autoreactive B cells, which produce autoantibodies. Patients with RA present elevated serum levels of B-cell activation factor associated with deregulated activation of NF-κB, characteristic of the pathogenesis of RA [36].

Since deregulated activation of NF-κB is implicated in multiple inflammatory diseases, targeting the NF-κB signaling pathway is an attractive approach for anti-inflammatory therapies. Several inhibitors have been developed to block different stages of NF-κB signaling. An increasing number of selective IKK inhibitors block the catalytic activity of IKK and prevent the phosphorylation of IκBα. Some known anti-inflammatory medications, such as aspirin and salicylate, also can inhibit IKK. Proteasome inhibitors, such as Velcade (also called Bortezomib and PS-341) and lactacystin, block the degradation of IκBα in the proteasome. Other inhibitors block the nuclear translocation of different subunits of NF-κB, such as tacrolimus (FK-506) and super-repressor IκBα. Finally, some drugs inhibit the DNA binding activity of NF-κB, such as glucocorticoids and peroxisome proliferator-activated receptor (PPARs) agonists.

Although significant progress has been made in the design of approaches to inhibit NF-κB, there are complexities to developing clinically available NF-κB-based drugs. Furthermore, although inhibition of NF-κB could be beneficial in the treatment of inflammatory diseases, there are obvious questions about the balance between efficacy and safety, as NF-κB function is also required to maintain normal immune responses and cell survival, and accumulation studies suggest that global inhibition of NF-κB signaling can cause serious side effects [36,37]. Therefore, a better understanding of the mechanisms underlying the pathological activation of NF-κB in different diseases is crucial for designing more specific and compelling therapeutic agents for treating inflammatory diseases. In addition, canonical and noncanonical NF-κB pathways also mediate RANK ligand-induced differentiation of monocytes/macrophages into bone-resorbing osteoclasts, the dysregulation of which contributes to the inflammatory bone loss associated with RA.

MTX, a disease modifier and immunosuppressant, is one of the most widely used drugs in treating RA. Under experimental conditions, rats with RA induced with Freund’s adjuvant and collagen type II, similar to the one used in this study (Figure 4), can also have their joint edema reduced [13]. One of the ethnomedical characteristics of *B. ternifolia* is its anti-inflammatory capacity in the face of different medical processes. Pharmacologically, this activity has been demonstrated in an atrial edema test with 12-O-Tetradecanoylphorbol-13-acetate (TPA), attributing the effect to terpene-type compounds such as ursolic acid, oleanolic acid, flavonoids derived from quercetin, the chlorogenic acid, and the scopoletin [9,10]. The coumarin was isolated from M1 of *B. ternifolia* in the present work, and this compound has been shown to be an excellent anti-inflammatory and antiarthritic in animal models; for example, the administration of 25, 50, and 100 mg/kg of this coumarin in rats with RA by Freund’s complete adjuvant decreases the size of the joint in a dose-dependent manner [14]. While bouvardin does not have data on its possible anti-inflammatory or antiarthritic effect, it has sufficient reports on its in vitro cytotoxic and anticancer capacity [38].

There is no information on the anti-inflammatory activity of ternifoliol or ternifolial (Figure 2a,b, respectively). However, it has been shown that chromone-type compounds, similar to ternifoliol and ternifolial, could modulate mechanisms associated with the inflammatory response and NF-κB expression [22].

## 4. Materials and Methods

### 4.1. Plant Material

Roots of *B. ternifolia* were collected in Huitzilac, Morelos, Mexico. (19°1′48″ N–99°15′56″ O); a specimen was deposited in the INAH Botanical Garden Herbarium and identified by biologists Margarita Avilez and Macrina Fuentes (INAH-MOR-2080).

### 4.2. General Experimental Procedures

Thin-layer chromatography (TLC) and high-performance liquid chromatography (HPLC) were used to monitor the chemical separation. TLC was performed using silica gel (60, F254) on 20 × 20 cm aluminum sheets (Merck KGaA, Darmstadt, Germany). The HPLC analysis procedure was carried out as described elsewhere [39] on a Waters apparatus equipped with Empower Pro software (Waters Corporation, USA). Mass spectrometry analysis was performed on a triple quadrupole mass spectrometer (Waters) using an electrospray Z-spray ion source in ESI positive mode. Source and desolvation temperatures were 150 and 400 °C, respectively. A cone voltage of 20 V and a capillary voltage of 2.5 kV were used. Nitrogen was employed as both a desolvation gas and cone gas. An MS scan was performed using argon gas as the collision gas (Agilent INC, Santa Clara, CA, USA). Compounds **2** and **4** NMR spectra were recorded on an Agilent DD2-600 at 600 MHz and compounds **1** and **3** in a Varian INOVA-400 instrument at 400 MHz for ^1^H NMR, NOESY, ^1^H-^1^H COSY, HSQC, and HMBC and DEPT in CDCl_3_ and CD_3_OD. Tetramethylsilane was used as an internal reference.

### 4.3. Extracts and Fractions from B. Ternifolia

The roots of the plant were dried at 40 °C in the dark. The dry material was ground (Pulvex SA de CV, Mexico City, Mexico) until obtaining particles between 1–5 mm and extracted by maceration with an ethanol/water mixture (60:40, Merck) for 20 h at room temperature. Subsequently, the extract was filtered and concentrated using a distillation process under reduced pressure and low temperature in a rotary evaporator Büchi-490 (Büchi, Postfach, Flawil, Switzerland) to yield the hydroalcoholic extract (BtEtOH). The BtEtOH extract was subjected to a fractionation process with water/ethyl acetate (1:3 *v*/*v*). The ethyl acetate and aqueous fractions were separated, concentrated, and lyophilized on a rotary evaporator to yield the BtAcOEt and BtAq fractions. The BtAcOEt fraction (6 g) was subjected to chromatographic fractionation in a glass column (1.9 × 40 cm) packed with silica gel 60 (70–230 mesh, Merck, Darmstadt, Germany), using a mixture of n-hexane/ethyl acetate/methanol and with a gradual increase in the polarity of the solvents, resulting in 74 fractions. The TLC analysis allowed them to be grouped into four fractions (M1, M2, M3, and M4).

### 4.4. Isolation and Identification of Compounds **1**, **2**, **3,** and **4**

The M3 fraction (5 g) was purified by successive open column normal phase chromatography using silica gel 60 as the stationary phase and a gradient of hexane/ethyl acetate as the mobile phase, collecting volumes of 15 mL to yield 48 fractions that were concentrated and pooled into seven subfractions (BM1, BM2, BM3, BM4, BM5, BM6, and BM7). The BM4 fraction (400 mg) was purified by normal phase open column chromatography (40 cm × 19mm) using silica gel 60 (15 g) as the stationary phase. A gradient of hexane/ ethyl acetate as the mobile phase was used to elute the column with an increase in polarity of 10% per 100 mL. Compound **1** resulted from this chromatographic process from the BM4 fraction, from which 48 fractions and 7 meetings were obtained; this new compound is called ternifoliol (**1**, 36.4 mg), is yellow in color, and dissolves in methanol. TLC showed a blue, fluorescent band when observed under UV light (λ = 365 nm). Fraction BM5 (600 mg) was purified by successive open column reverse phase chromatography using RP-18 silica gel (6 g) as the stationary phase and a water/acetonitrile gradient as the mobile phase. Seventy-eight fractions were acquired. Compound **2** was identified in the BM5 fraction and turned out to be a new molecule, which we named ternifolial (**2**, 21.6 mg). Compound **2** is yellow and dissolves in methanol. TLC showed a blue, fluorescent band when observed under UV light λ = 365 nm. The BM6 fraction (400 mg) was purified by successive open column normal phase chromatography (40 cm × 19 mm) using silica gel (15 g) as the stationary phase. A hexane/ethyl acetate gradient was the mobile phase used to elute the column with an increase in polarity of 5% per 15 mL. In fraction BM6, bouvardin (3, 26 mg) was identified. Bouvardin is yellow and dissolves in methanol. Bouvardin is revealed with ninhydrin on TLC and shows purple spots. Fraction M2 (10 g) was purified by normal phase chromatography on an open column with silica gel 60 following the separation procedure described for compound **1**. In fraction M2, scopoletin (4, 4.18 mg) was identified. Scopoletin is white and dissolves in methanol. TLC showed a blue, fluorescent band when observed under UV light (λ = 365 nm).

### 4.5. Raw-Blue™ Cells Culture Experiments

Raw-blue™ cells were cultured following the manufacturer’s recommendations (InvivoGen, San Diego, CA, USA). A different treatment was added to each culture well; at 1 h of incubation, LPS (1 µg/mL) (Merck KGaA, Darmstadt, Germany) was added to activate NF-κB. The microplate was incubated for 24 h at 37 °C in 5% CO2. We added 150 µL of QUATI-blue™ (InvivoGen, San Diego, CA, USA) to 50 µL of medium supernatant to measure the secreted alkaline phosphatase activity [20]. At the incubation time at 37 °C, the absorbance at 630 nm was measured. The culture medium was used as basal control and stimulus supernatant with LPS as 100% activity. The inhibition activity (%) of NF-κB was calculated using the following equation:

$$\mathrm{IA}=\frac{A-B}{A}\times 100$$

IA: Inhibition activity (%)

A: Absorbance of 100% activity;

B: Absorbance of the treatment.

Methotrexate (80 ng) was used as a positive control of NF-κB inhibitor activity. All experiments were performed four times with one repetition each.

### 4.6. Evaluation of the Antiarthritic Effect In Vivo of Bouvardia Ternifolia

Female ICR mice were used (34–42 g average), obtained from the animal center of the Health Research Coordination of the Siglo XXI Medical Center, Mexico City, in strict accordance with Mexican regulations for the use of experimental animals (NOM-062ZOO-1999). In addition, the protocol was registered with the institutional research and ethics committee (R-2010-1701-57). The rheumatoid arthritis model was established by injecting mice four times (on days 0, 7, 14, and 21) with 10 μL of collagen type II 1 mg/mL (Merck KGaA) suspended in Freund’s Complete Adjuvant (CFA, Sigma-Aldrich). The immunization was carried out under surgical anesthesia with sodium pentobarbital (60 mg/kg i.p, Aranda Lab, Mexico City, Mexico) at the base of the tail.

The administration of the treatments began on day 15. Each group (n = 7) was administered for 15 days, with corresponding treatment (Table 2). Throughout the period, the development of joint inflammation was measured, recording the diameter of the patellofemoral joint with an electronic micrometer (Mitutoyo S.A de C.V. Naucalpan de Juárez, Edo. de Méx. México). At the end of the experimental period, the animals were ethically euthanized to obtain patellofemoral joints and kidneys. Each of these organs was homogenized in 1× PBS with 0.1% protease inhibitor (phenyl-methyl-sulfonyl fluoride, PMFS from Merck KGaA, Darmstadt, Germany); the homogenate was centrifuged for 5 min at 33,000× *g*. The supernatant was stored at −70 °C until analysis. The cytokine concentrations were quantified by ELISA (IL-1β, IL-6, IL-10, IL-17, and TNF-α) following the manufacturer’s instructions (Becton, Dickinson and Co. Franklin Lakes, NJ, USA).

## 5. Conclusions

In conclusion, the extracts and fractions of *B. ternifolia* were characterized, identifying two novel compounds defined as chromones: ternifoliol and ternifolial; in addition to bouvardin and scopoletin. The fractions M1, M2, M3, M4 and the compounds isolated from the Bouvardia tenifolia root inhibited the expression of NF-κB. Furthermore, in the in vivo systemic model of experimental rheumatoid arthritis induced with CFA + type II collagen, all the fractions evaluated (particularly the aqueous fraction) had immunomodulatory effects on proinflammatory and anti-inflammatory cytokines, both in the kidneys and in the synovial joints.

Two new chromone-type compounds called ternifoliol and ternifolial that have anti-inflammatory activity on the NF-κB pathway were isolated.

## Figures and Tables

**Table 1:** ^1^H-NMR and ^13^C-NMR spectroscopy data of 1 (CD_3_OD, 400 MHz) and 2 (DCl_3_, 600 MHz).

Position	^1^H (J in Hz) 1	^13^C	^1^H (J in Hz) 2	^13^C
1		72.9		133.6
2	3.81 (1H, s, br)	83.9	4.56 (1H, dd, 7.3, 8.8)	119.3
3	a) 3.20 (1H, dd, 3.6, 13.5)	23.6	a) 3.41 (1H, dd, 7.3, 13.9)	37.2
3a	b) 2.72 (1H, dd, 3.6, 13.5)	115.9	b) 2.74 (1H, dd, 7.3, 13.9)	56.7
4		147.9		204.9
5		120.6		132.7
6	7.86 (H dd, 0.7, 9.1)	123.9		137.6
6a	7.13 (1H, d, 9.1)	125.2		127.57
7		149.4	6.21 (1H, d, 10.27)	154.1
8		105.7		113.4
9	6.90 (1H, s)	112.7	7.28 (1H, s)	112.2
10		152.6		144.7
10a		122.7		127.53
11		169.2	6.21 (1H, d, 10.27)	170.6
12		25.9		25.6
13	1.38 (3H, s)	26.4	1.44 (1H, d, 16.14)	17.7
OCH_3_	1.44 (3H, s)	52.42	1.44 (1H, d, 16.14)	133.6

**Table 2:** Groups and experimental treatments with collagen type II and CFA.

Group	Oral Treatment	Doses
Baseline	Water + tween 1%	100 µL/10 g
Veh	Water + tween 1% + collagen type II + CFA *	1 mg/mL
Experimental	Methotrexate	25 mg/kg every 5 days
	BtAq	25 mg /kg for 15 days
	BtAcOEt	
	M1	
	M2	
	M4	

* Lipopolysaccharide; Baseline = healthy mice; Veh = animals with collagen type II + CFA but without treatment; BtAq = aqueous extract from *B. ternifolial*; BtAcOEt = ethyl acetate extract; M1; M2; M3; M4.

## Data Availability

Not applicable.

## Supplementary Materials

The following supporting information can be downloaded at: https://www.mdpi.com/article/10.3390/plants12010001/s1. Figure S1. Spectrum UV of ternifoliol (**1**); Figure S2. ^1^H NMR (CD_3_OD, 400 MHz) of ternifoliol (**1**); Figure S3. ^13^C NMR (CD_3_OD, 100 MHz) of ternifoliol (**1**); Figure S4. ^13^C (DEPT) NMR (CD_3_OD, 100 MHz) of ternifoliol (**1**); Figure S5. ^1^H-^1^H COSY NMR (CD_3_OD, 400 MHz) of ternifoliol (**1**); Figure S6. ^1^H-^13^C (HSQC) NMR (CD_3_OD, 400 MHz) of ternifoliol (**1**); Figure S7. ^1^H-^13^C (HMBC) NMR (CD_3_OD, 400 MHz) of ternifoliol (**1**); Figure S8. Mass spectrum (MS) of ternifoliol (**1**); Figure S9. Spectrum UV of ternifolial (**2**)., Figure S10. ^1^H NMR (CDCl_3_, 600 MHz) of ternifolial (2); Figure S11. ^13^C NMR (CDCl_3_, 150 MHz) of ternifolial (**2**); Figure S12. ^1^H-^1^H COSY NMR (CDCl_3_, 600 MHz) of ternifolial (**2**); Figure S13. ^1^H-^1^H TOCSY NMR (CDCl_3_, 600 MHz) of ternifolial (**2**); Figure S14. ^1^H-^1^H NOESY NMR (CDCl_3_, 600 MHz) of ternifolial (**2**); Figure S15. ^1^H-^13^C (HSQC) NMR (CDCl_3_, 600 MHz) of ternifolial (**2**); Figure S16. ^1^H-^13^C (HMBC) NMR (CDCl_3_, 600 MHz) of ternifolial (**2**); Figure S17. Mass spectrum (MS) of ternifolial (**2**); Figure S18. Spectrum UV of bouvardin (**3**); Figure S19. ^1^H NMR (CD_3_OD, 400 MHz) of bouvardin (**3**); Figure S20. ^13^C NMR (CD_3_OD, 100 MHz) of bouvardin (**3**); Figure S21. ^13^C (DEPT) NMR (CD_3_OD, 100 MHz) of bouvardin (**3**); Figure S22. ^1^H-^1^H COSY NMR (CD_3_OD, 400 MHz) of bouvardin (**3**); Figure S23. ^1^H-^1^H NOESY NMR (CD_3_OD, 400 MHz) of bouvardin (**3**); Figure S24. ^1^H-^13^C (HMBC) NMR (CD_3_OD), 400 MHz) of bouvardin (**3**); Figure S25. Mass spectrum (MS) of bouvardin (**3**); Figure S26. Spectrum UV of scopoletin (**4**); Figure S27. ^1^H NMR (CDCl_3_, 600 MHz) of scopoletin (**4**); Figure S28. ^13^C NMR (CDCl_3_, 150 MHz) of scopoletin (**4**); Figure S29. ^13^C (DEPT) NMR (CDCl_3_, 150 MHz) of scopoletin (**4**); Table S1: ^13^C NMR (CD_3_OD, 100 MHz) of bouvardin (**3**), ^1^H-^13^C (HSQC) NMR (CD_3_OD, 400 MHz) and ^13^C NMR (CDCL_3,_ Bates et al., 1983). Figure S30. Infrared data of ternifoliol (**1**); Figure S31. Infrared data of ternifolial (**2**).
