# Supplementary material for: New Chromones from Bouvardia ternifolia (Cav.) Schltdl with Anti-Inflammatory and Immunomodulatory Activity
Source: Plants (Basel). 2022 Dec 20;12(1):1. doi: 10.3390/plants12010001 (PMC9823701; doi:10.3390/plants12010001)
Supplement: Supplementary file 1 [file plants-12-00001-s001.zip › plants-2039050-supplementary.pdf]

plants

New chromones from *Bouvardia ternifolia* (Cav.) Schltdl with anti-inflammatory and immunomodulatory activity.

Yury Maritza Zapata Lopera [1,2], Enrique Jiménez-Ferrer [1,*], Maribel Herrera-Ruiz [1], Alejandro Zamilpa [1], Manasés González-Cortazar [1], Gabriela Rosas-Salgado [3], Mayra Alejandra Santillán-Urquiza [1], Gabriela Trejo-Tapia [2] and Antonio Ruperto Jiménez-Aparicio [2,*]

1 Centro de Investigación Biomédica del Sur, Instituto Mexicano del Seguro Social, Xochitepec 62790, Morelos, Mexico
2 Instituto Politécnico Nacional, Centro de Desarrollo de Productos Bióticos, Yautepec 62730, Morelos, Mexico
3 Facultad de Medicina, Universidad Autónoma del Estado de Morelos, Cuernavaca 62209, Morelos, Mexico
* Correspondence: enriqueferrer_mx@yahoo.com (E.J.-F.); aaparici@ipn.mx (A.R.J.-A.); Tel.: +52-7771355563 (E.J.-F.)

Content

Figure S1. Spectrum UV of ternifoliol (**1**)

Figure S2. ^{1}H NMR (CD_3OD, 400 MHz) of ternifoliol (**1**)

Figure S3. ^{13}C NMR (CD_3OD, 100 MHz) of ternifoliol (**1**)

Figure S4. ^{13}C (DEPT) NMR (CD_3OD, 100 MHz) of ternifoliol (**1**)

Figure S5. ^{1}H-^{1}H COSY NMR (CD_3OD, 400 MHz) of ternifoliol (**1**)

Figure S6. ^{1}H-^{13}C (HSQC) NMR (CD_3OD, 400 MHz) of ternifoliol (**1**)

Figure S7. ^{1}H-^{13}C (HMBC) NMR (CD_3OD, 400 MHz) of ternifoliol (**1**)

Figure S8. Mass spectrum (MS) of ternifoliol (**1**)

Figure S9. Spectrum UV of ternifolial (**2**)

Figure S10. ^{1}H NMR ($CDCl_3$, 600 MHz) of ternifolial (**2**)

Figure S11. ^{13}C NMR ($CDCl_3$, 150 MHz) of ternifolial (**2**)

Figure S12. ^{1}H-^{1}H COSY NMR ($CDCl_3$, 600 MHz) of ternifolial (**2**)

Figure S13. ^{1}H-^{1}H TOCSY NMR ($CDCl_3$, 600 MHz) of ternifolial (**2**)

Figure S14. ^{1}H-^{1}H NOESY NMR ($CDCl_3$, 600 MHz) of ternifolial (**2**)

Figure S15. ^{1}H-^{13}C (HSQC) NMR ($CDCl_3$, 600 MHz) of ternifolial (**2**)

Figure S16. ^{1}H-^{13}C (HMBC) NMR ($CDCl_3$, 600 MHz) of ternifolial (**2**)

Figure S17. Mass spectrum (MS) of ternifolial (**2**)

Figure S18. Spectrum UV of bouvardin (**3**)

Figure S19. ^{1}H NMR (CD_3OD, 400 MHz) of bouvardin (**3**)

Figure S20. ^{13}C NMR (CD_3OD, 100 MHz) of bouvardin (**3**)

Figure S21. ^{13}C (DEPT) NMR (CD_3OD, 100 MHz) of bouvardin (**3**)

Figure S22. ^{1}H-^{1}H COSY NMR (CD_3OD, 400 MHz) of bouvardin (**3**)

Figure S23. ^{1}H-^{1}H NOESY NMR (CD_3OD, 400 MHz) of bouvardin (**3**)

Figure S24. ^{1}H-^{13}C (HMBC) NMR (CD_3OD), 400 MHz) of bouvardin (**3**)

Figure S25. Mass spectrum (MS) of bouvardin (**3**)

Figure S26. Spectrum UV of Scopoletin (**4**)

Figure S27. ^{1}H NMR ($CDCl_3$, 600 MHz) of scopoletin (**4**)

Figure S28. ^{13}C NMR ($CDCl_3$, 150 MHz) of scopoletin (**4**)

Figure S29. ^{13}C (DEPT) NMR ($CDCl_3$, 150 MHz) of scopoletin (**4**)

Figure S30. IF spectroscopy ternifoliol

Figure S31. IF spectroscopy ternifolial

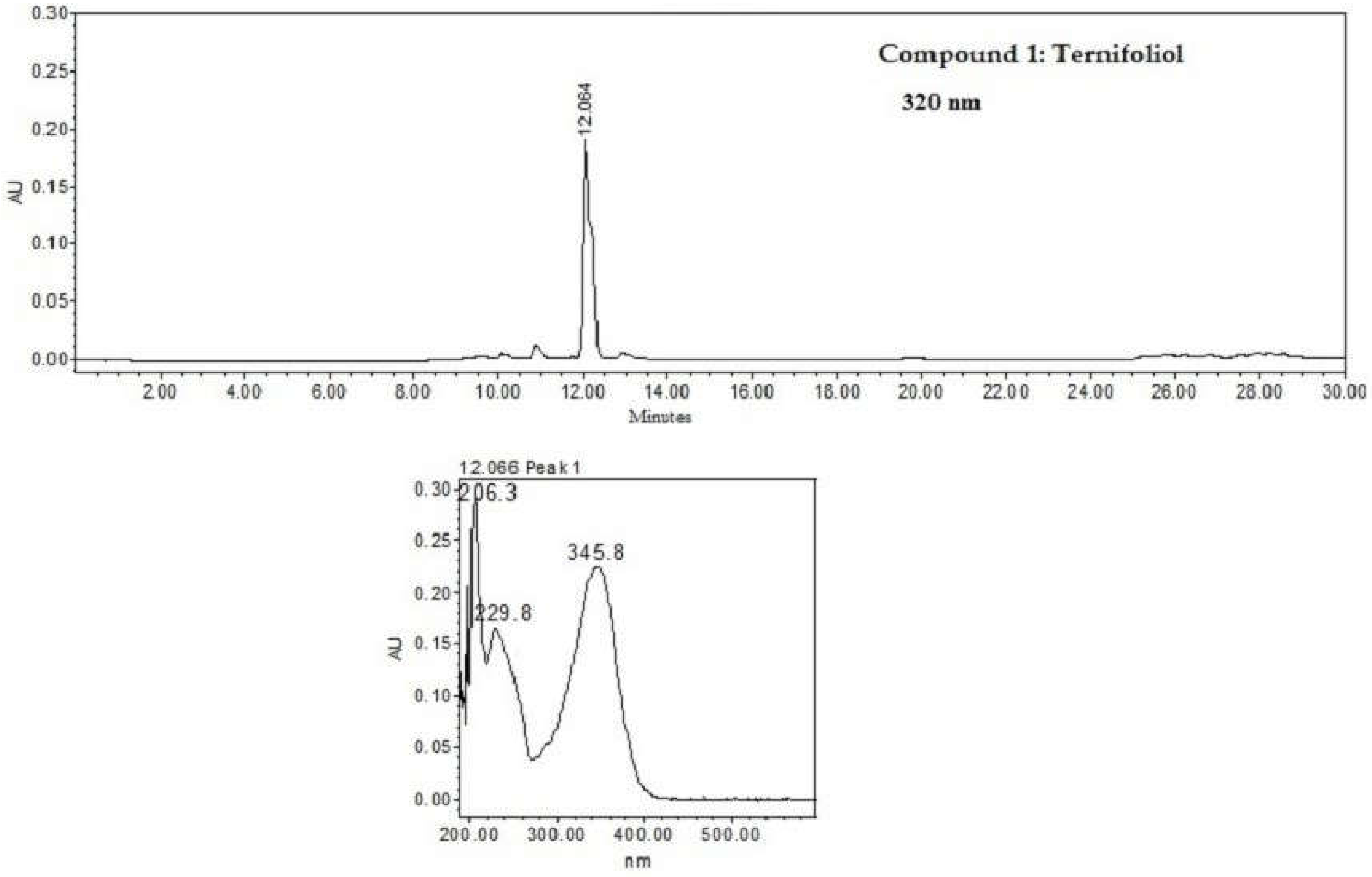

Figure S1. Spectrum UV of ternifoliol (**1**)

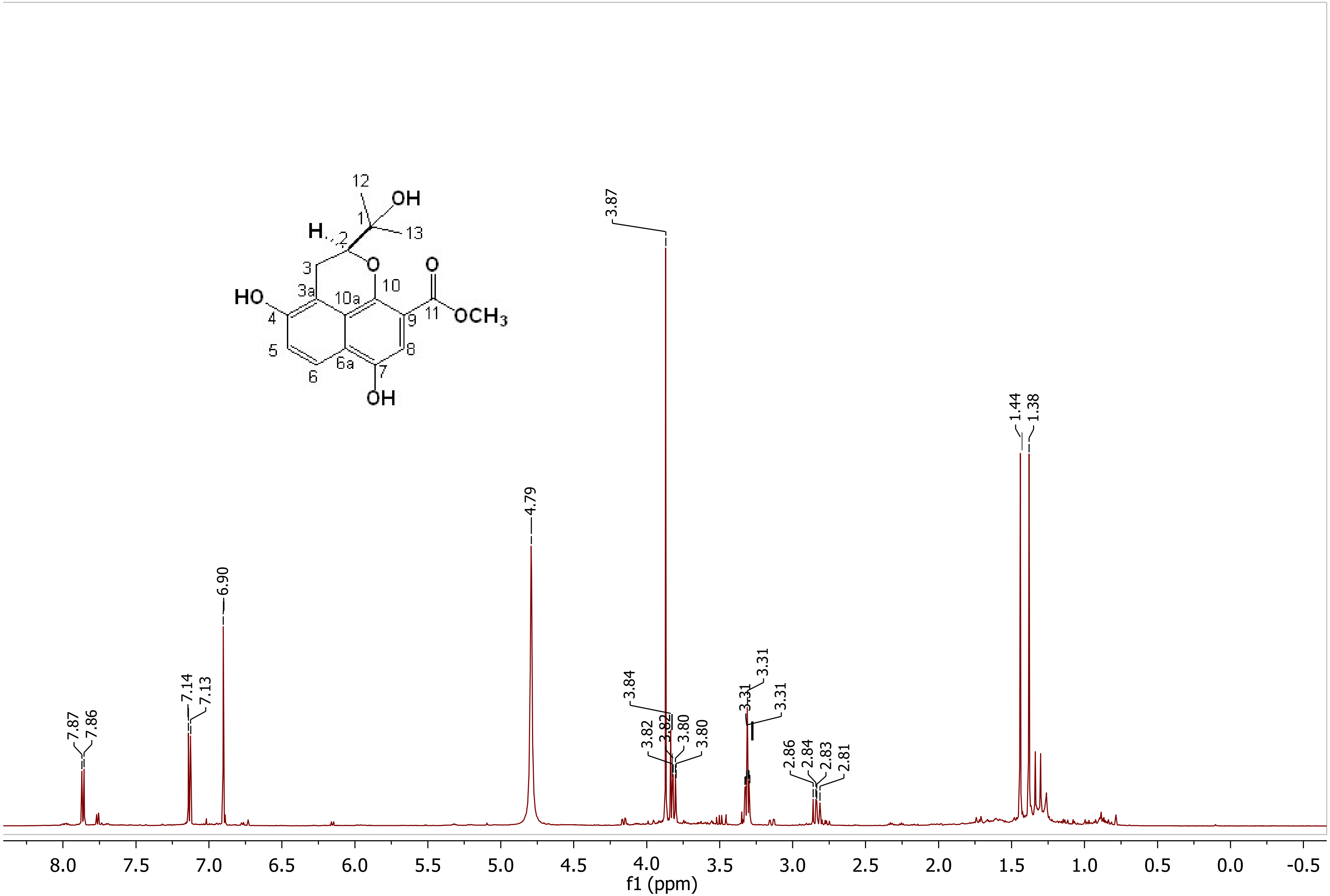

Figure S2. ^{1}H NMR ($CDCl_3$, 400 MHz) of ternifoliol (**1**)

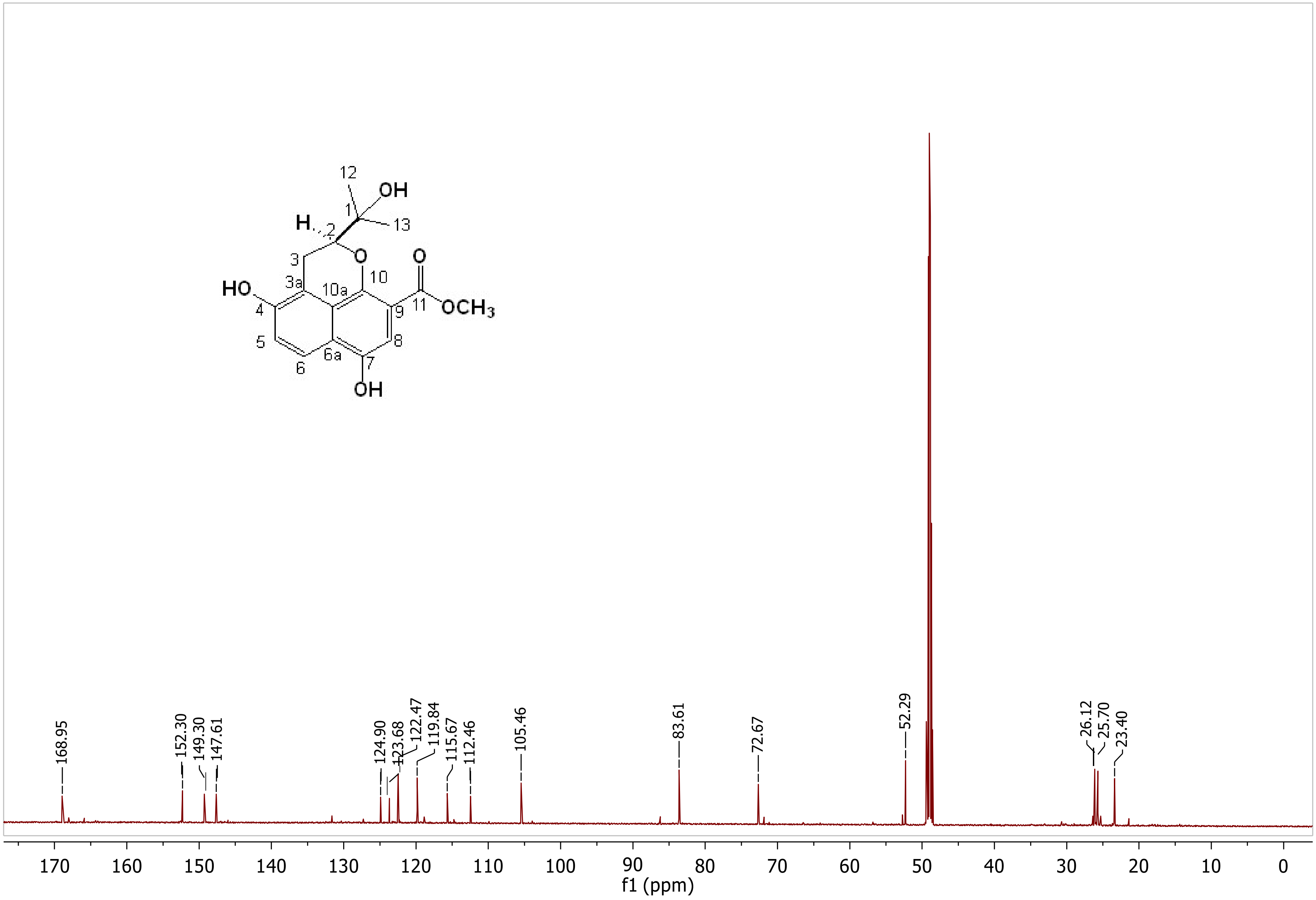

Figure S3. ^{13}C NMR (CD_3OD, 125 MHz) of ternifoliol (**1**)

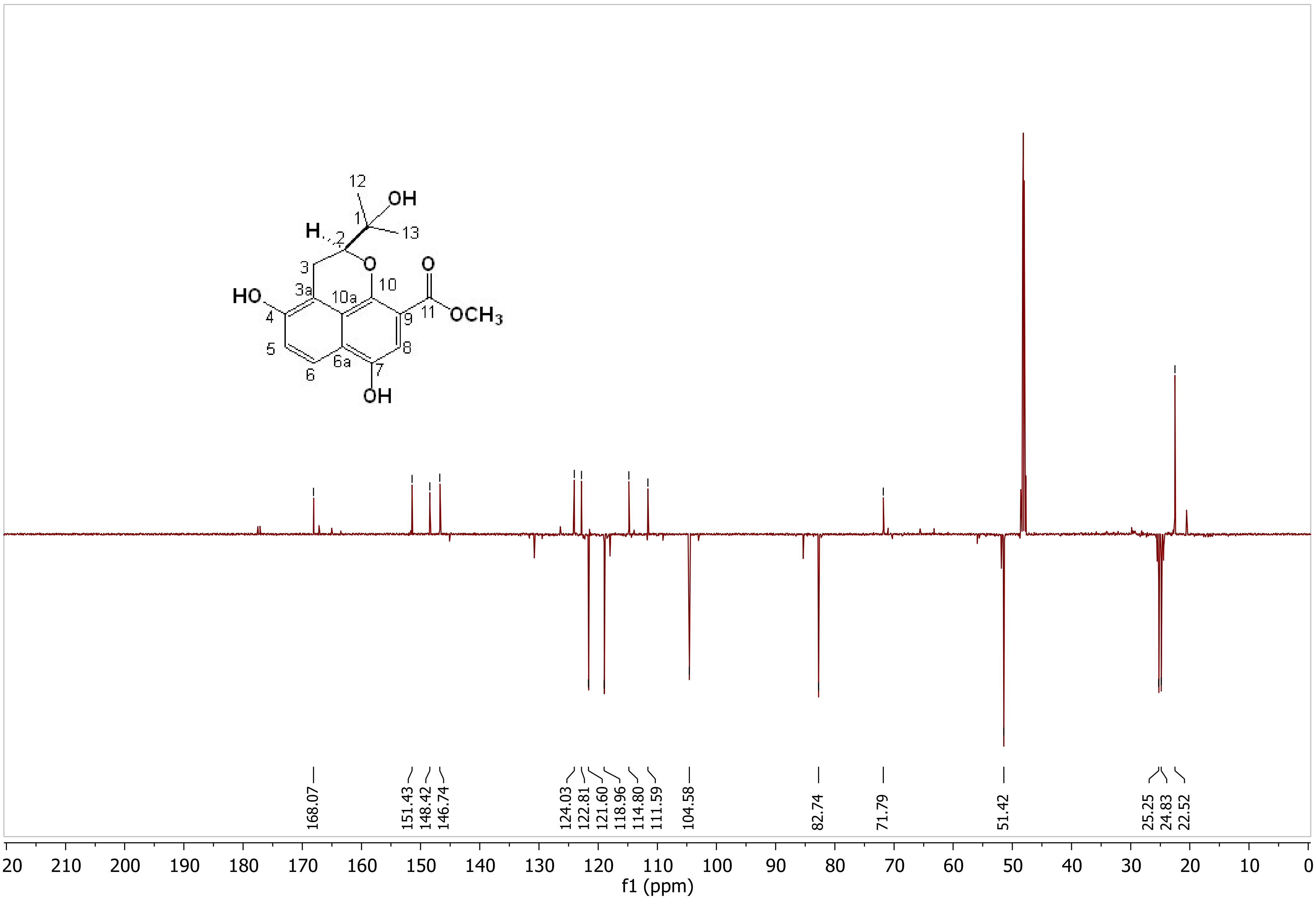

Figure S4. ^{13}C DEPT (CD_3OD, 125 MHz) of ternifoliol (**1**)

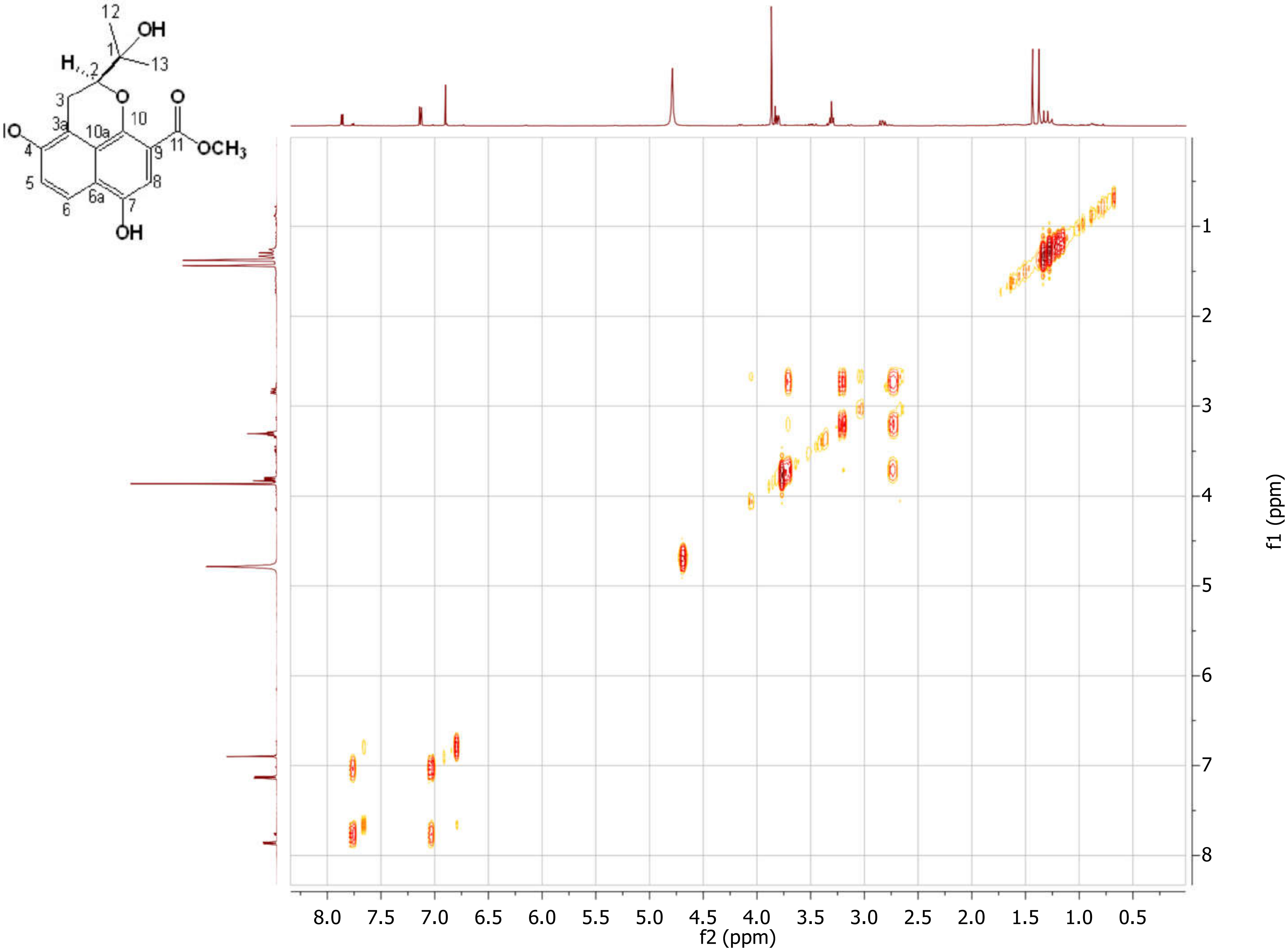

Figure S5. ^{1}H-^{1}H COSY NMR (CD_3OD, 400 MHz) of ternifoliol (**1**)

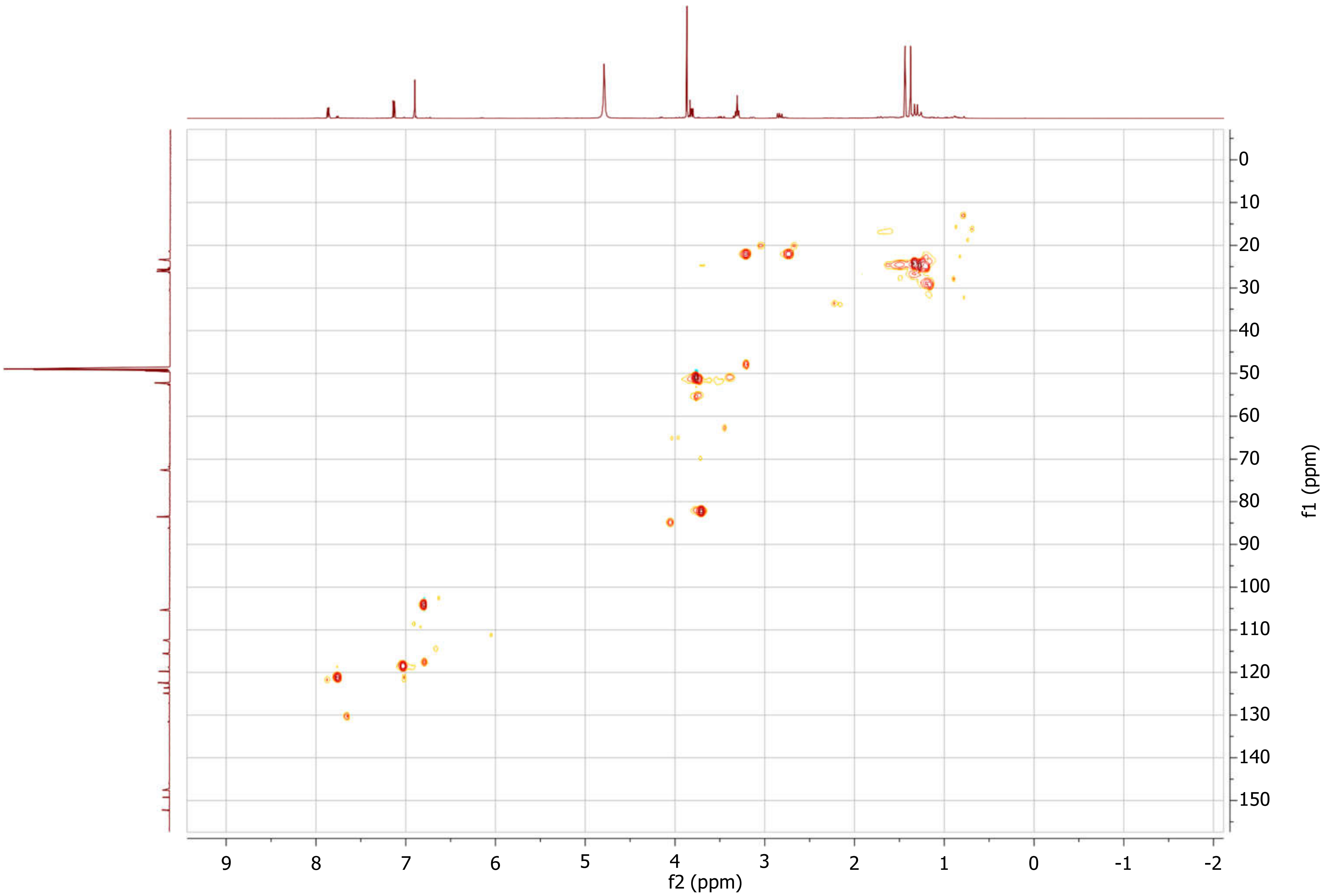

Figure S6. ^{1}H-^{13}C (HSQC) NMR (CD_3OD, 400 MHz) of ternifoliol (**1**)

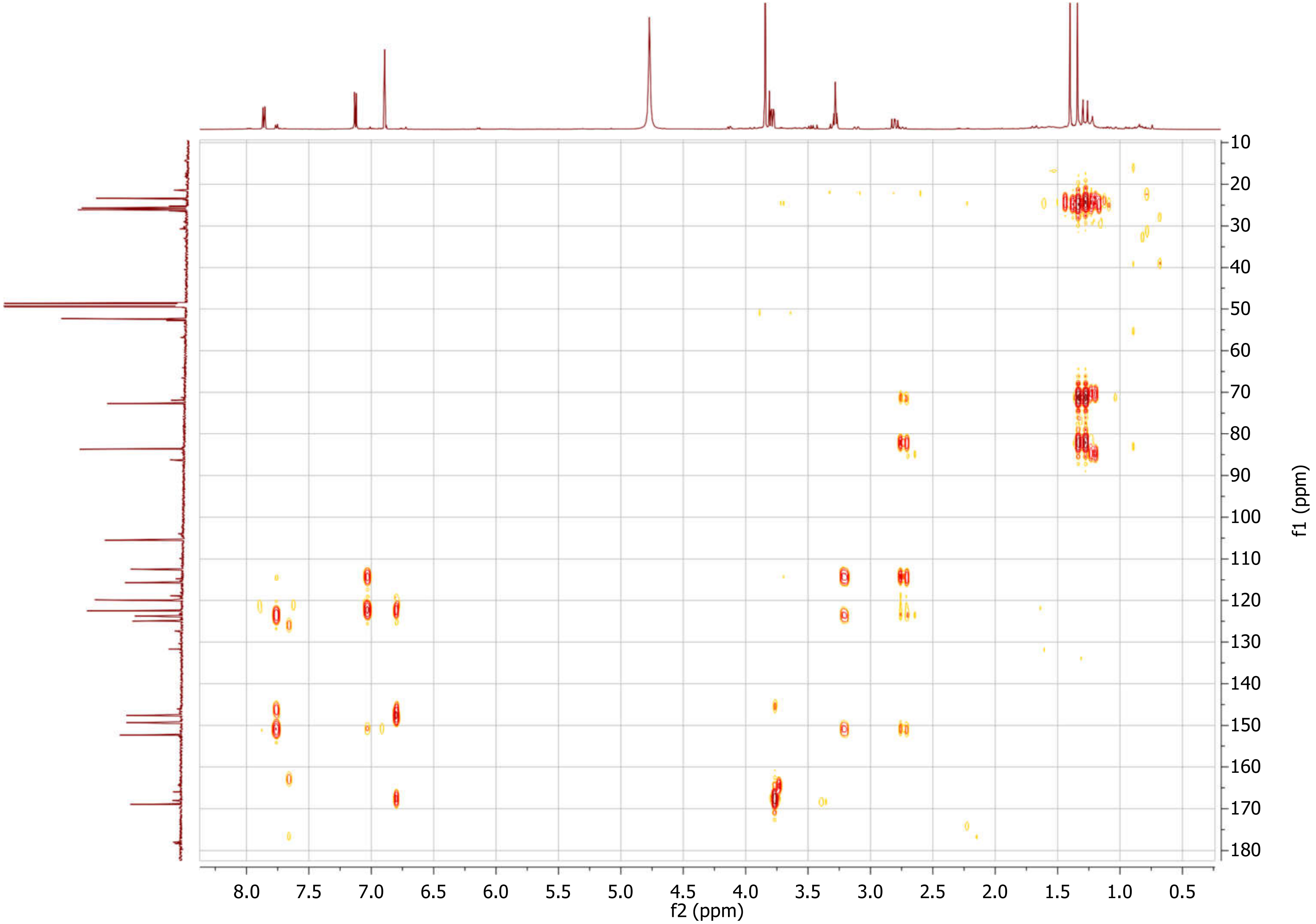

Figure S7. ^{1}H-^{13}C (HMBC) NMR (CD_3OD, 400 MHz) of ternifoliol (**1**)

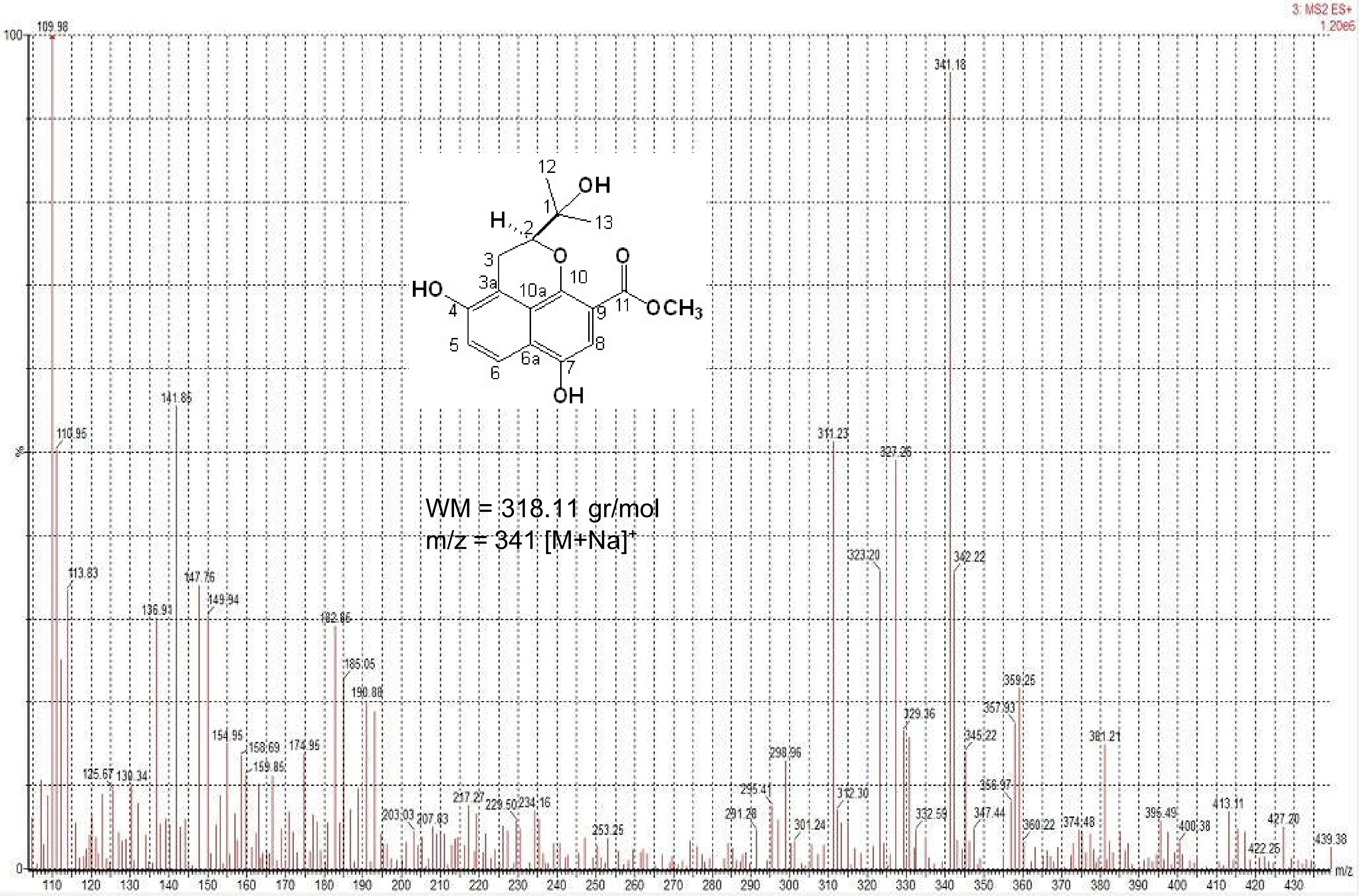

Figure S8. Mass spectrum (MS) of ternifoliol (**1**)

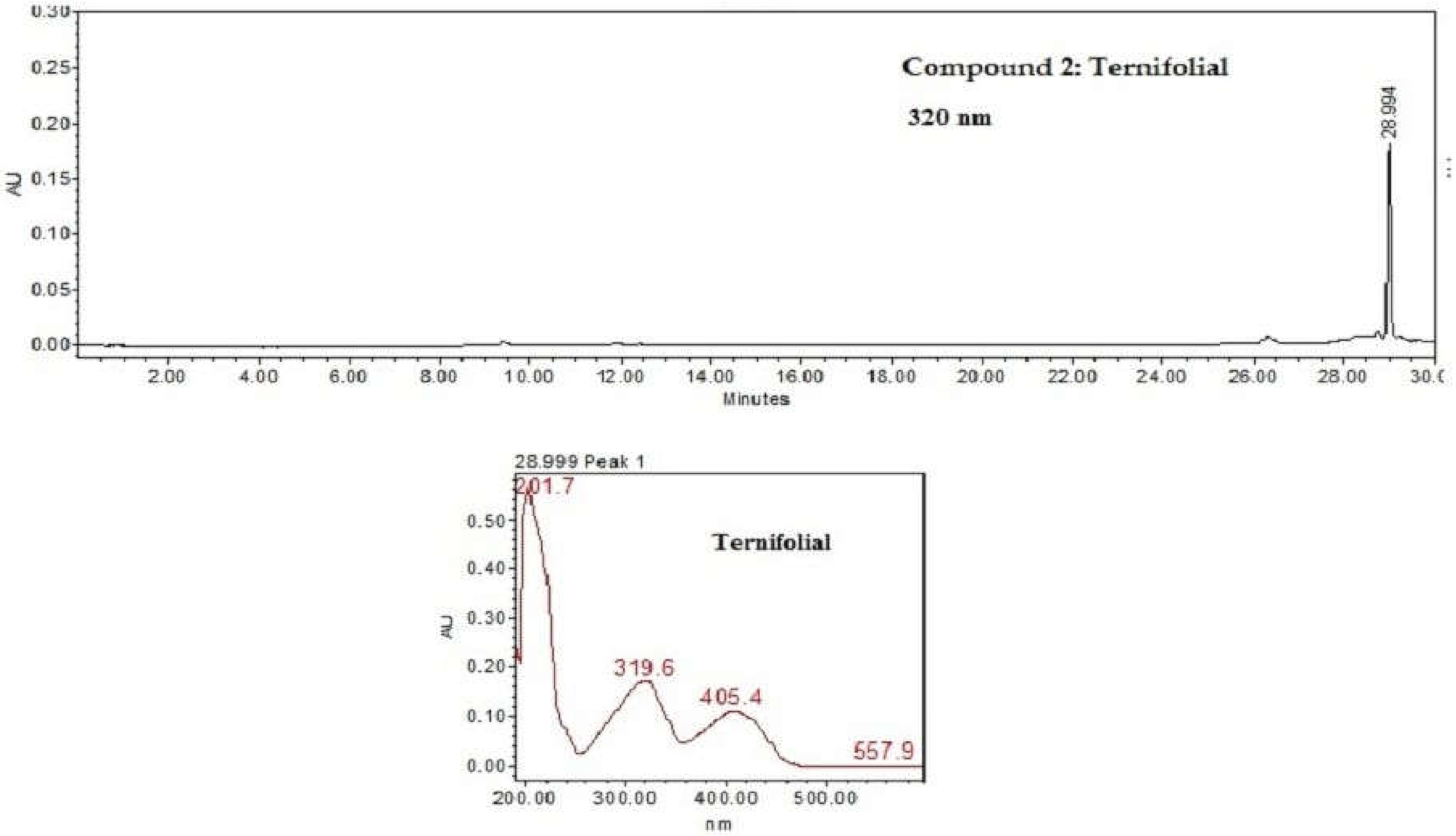

Figure S9. Spectrum UV of ternifolial (**2**)

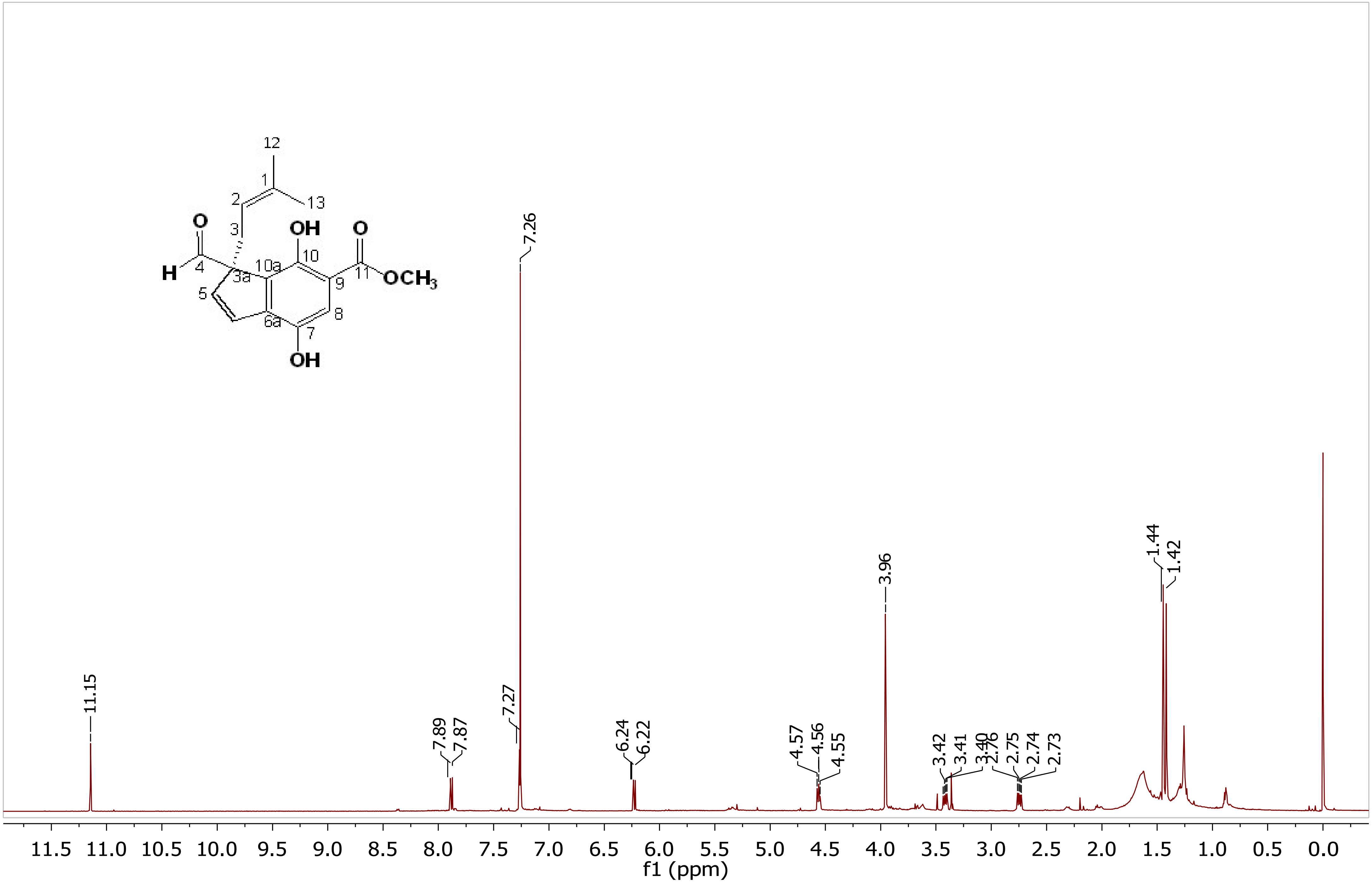

Figure S10. ^{1}H NMR ($CDCl_3$, 600 MHz) of ternifolial (**2**)

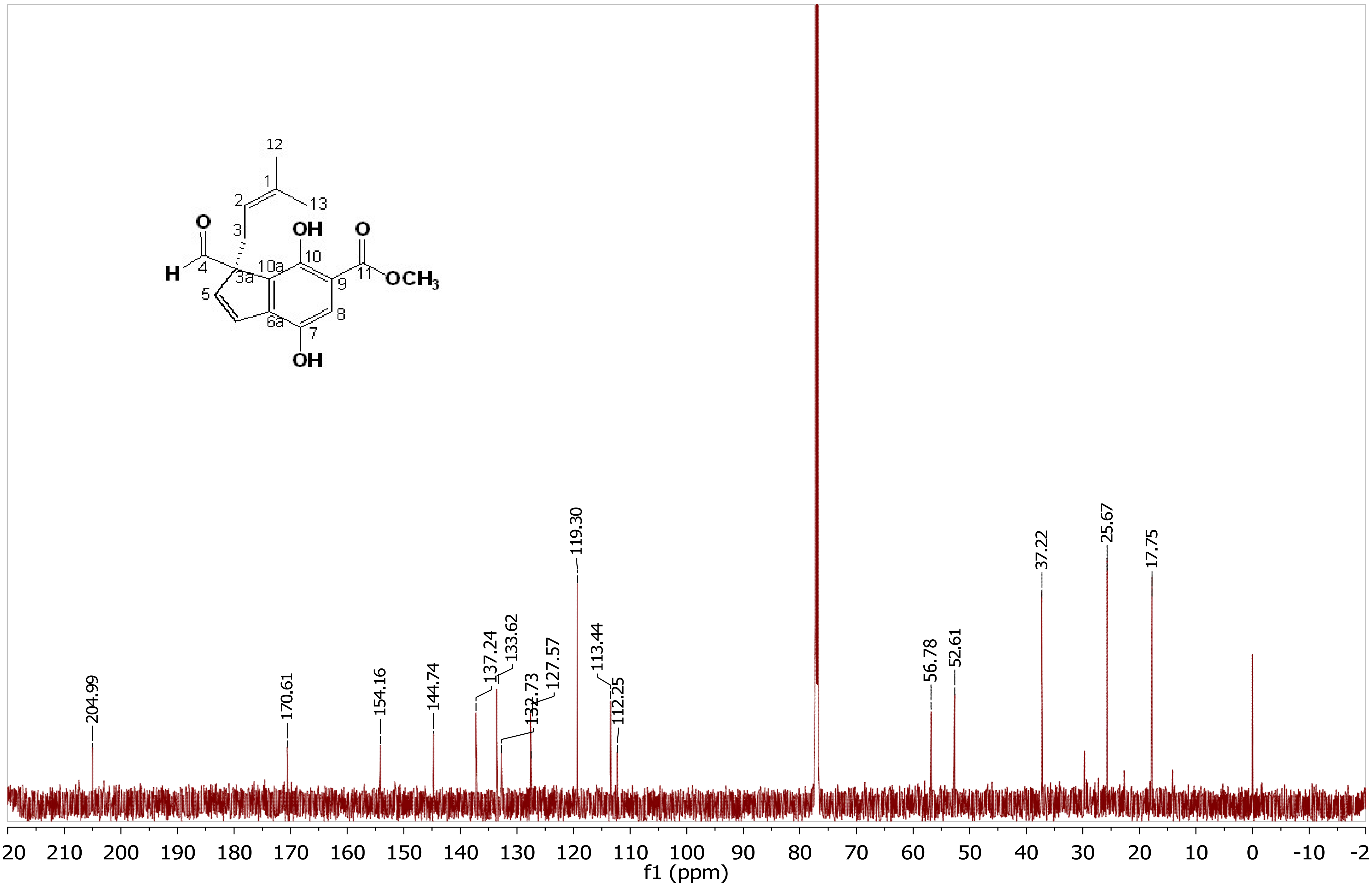

Figure S11. ^{13}C NMR ($CDCl_3$, 150 MHz) of ternifolial (**2**)

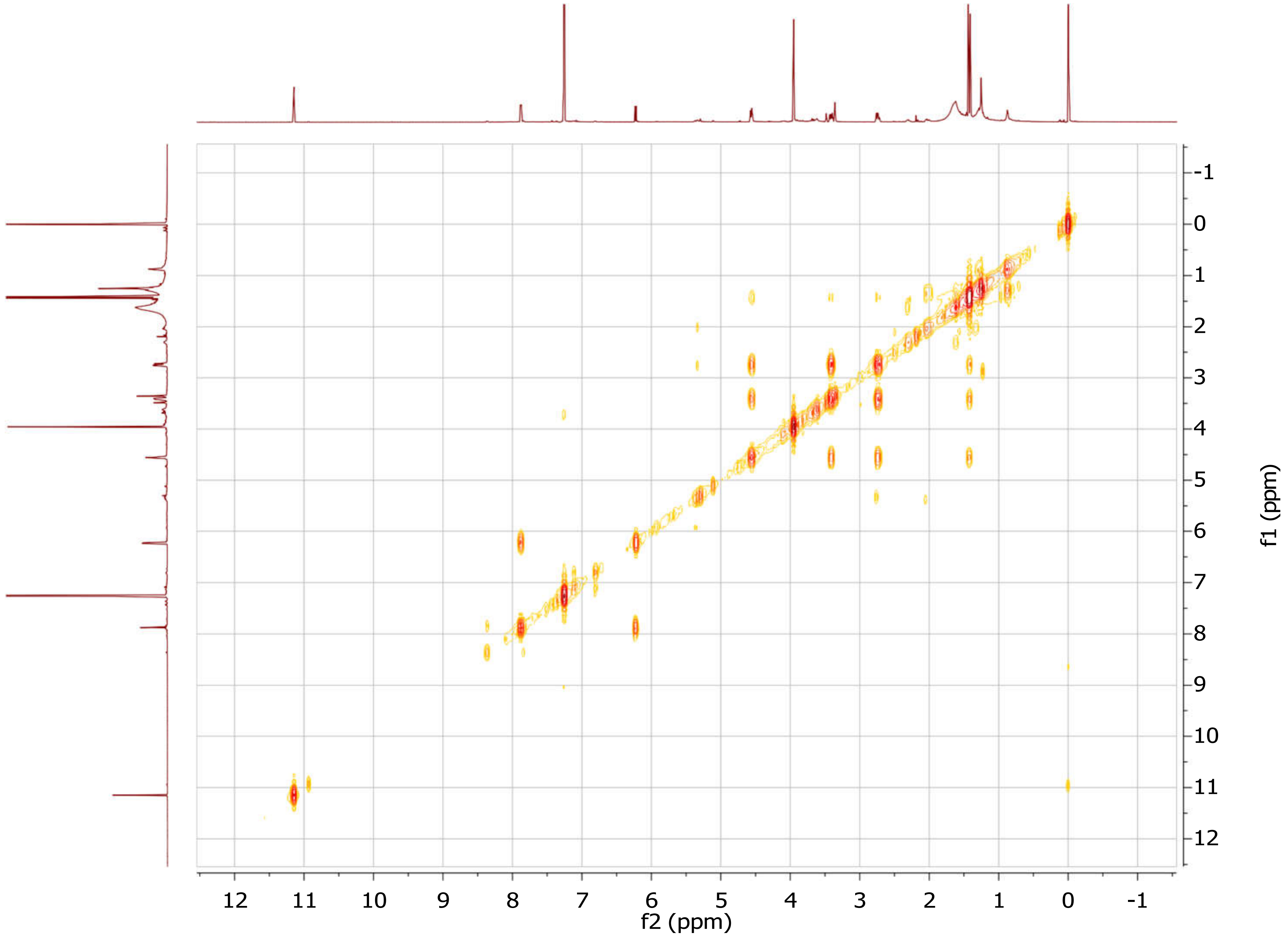

Figure S12. ^{1}H-^{1}H COSY NMR ($CDCl_3$, 600 MHz) of ternifolial (**2**)

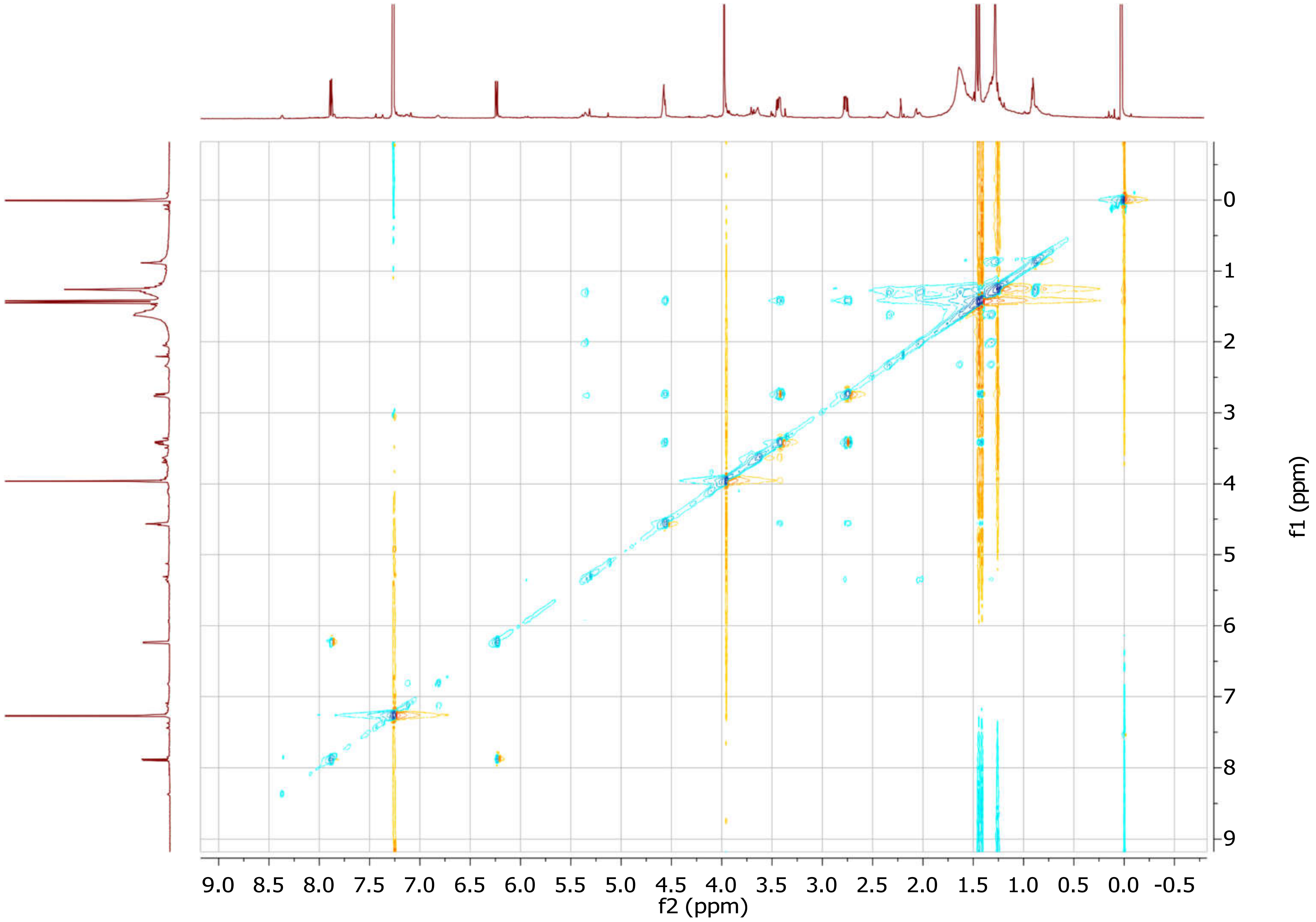

Figure S13. ^1H-^1H TOCSY NMR ($CDCl_3$, 600 MHz) of ternifolial (**2**)

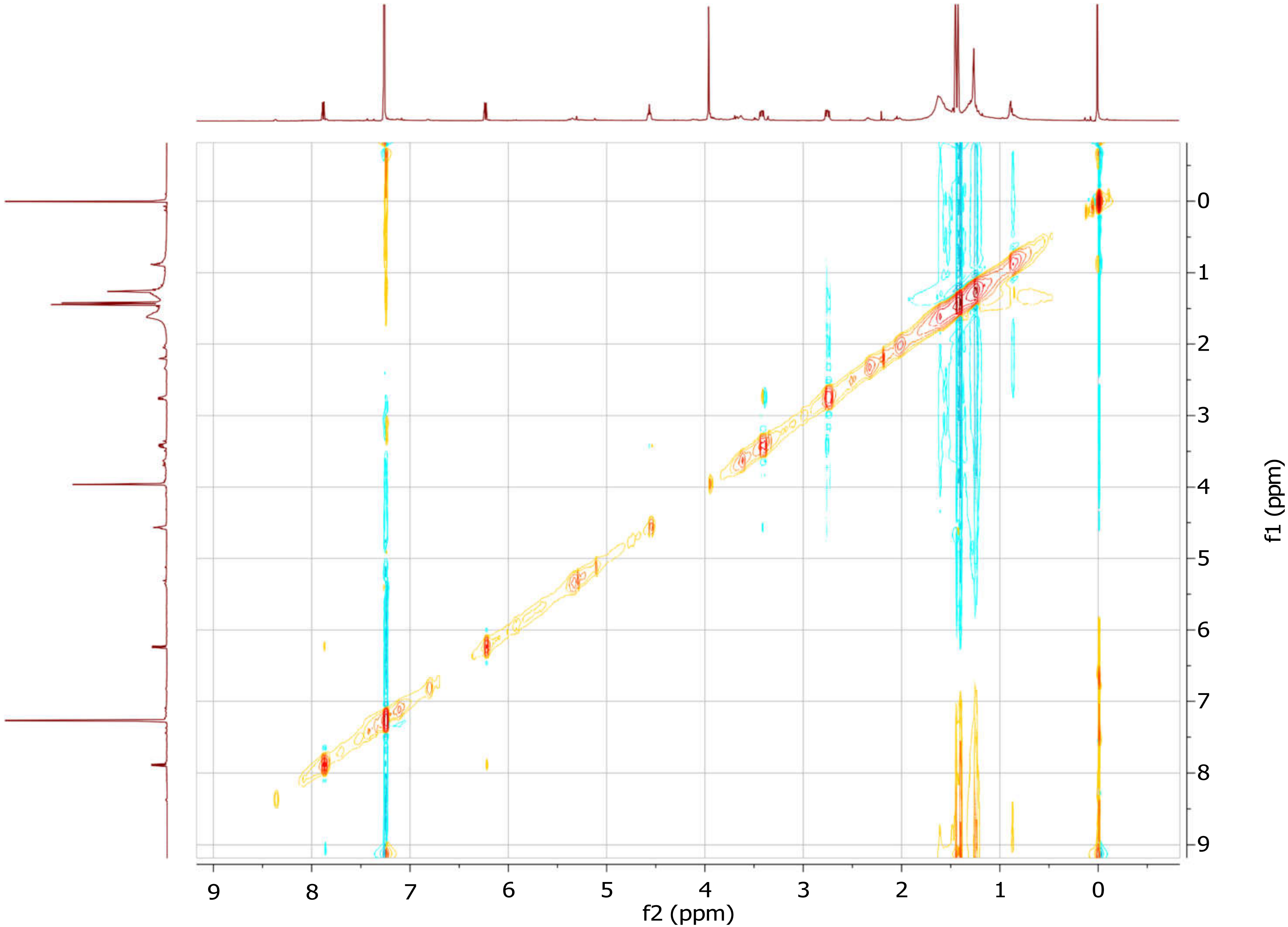

Figure S14. ^{1}H-^{1}H NOESY NMR ($CDCl_3$, 600 MHz) of ternifolial (**2**)

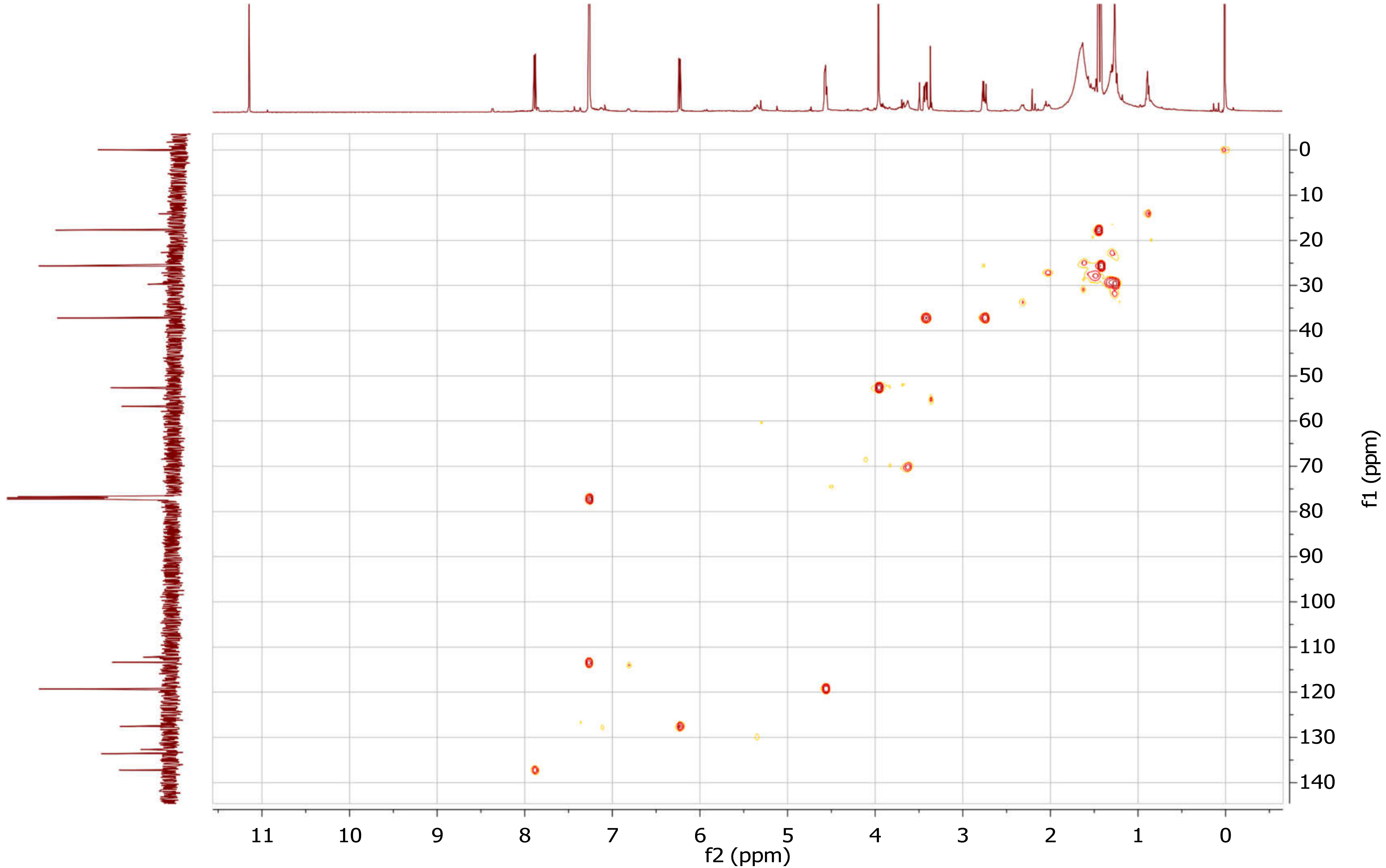

Figure S15. ^{1}H-^{13}C (HSQC) NMR ($CDCl_3$, 600 MHz) of ternifolial (**2**)

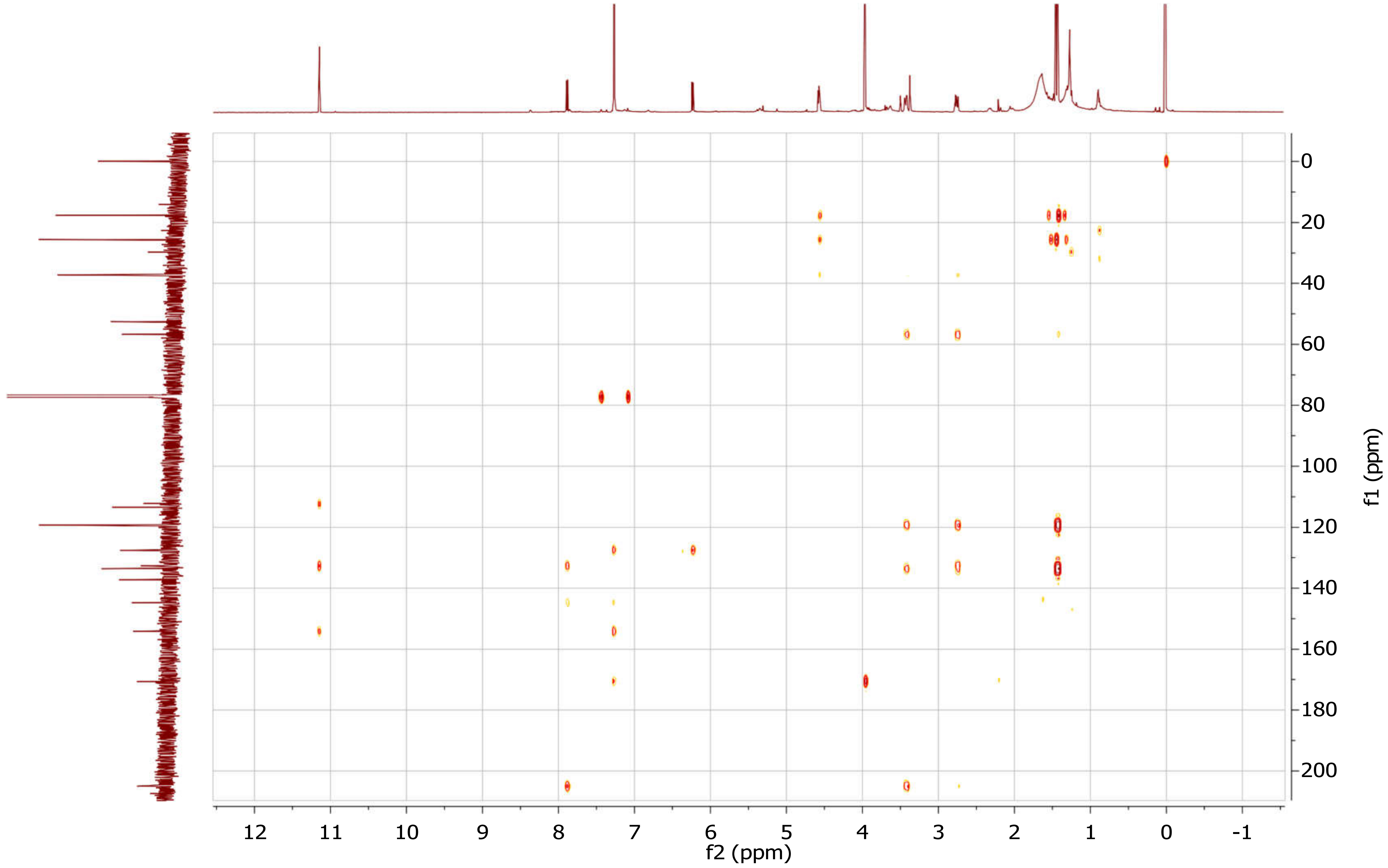

Figure S16. ^{1}H-^{13}C (HMBC) NMR ($CDCl_3$, 600 MHz) of ternifolial (**2**)

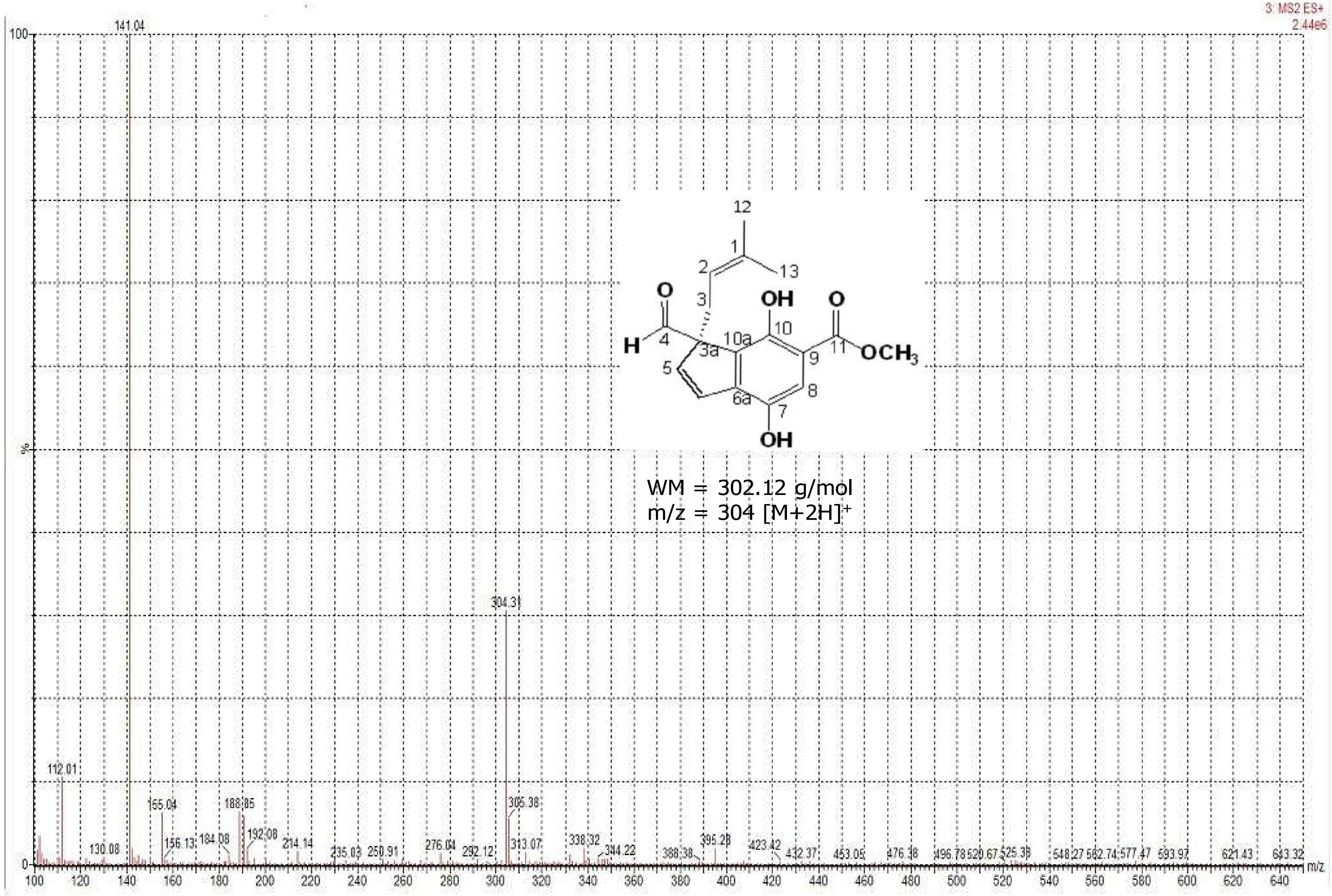

Figure S17. Mass spectrum (MS) of ternifolial (**2**)

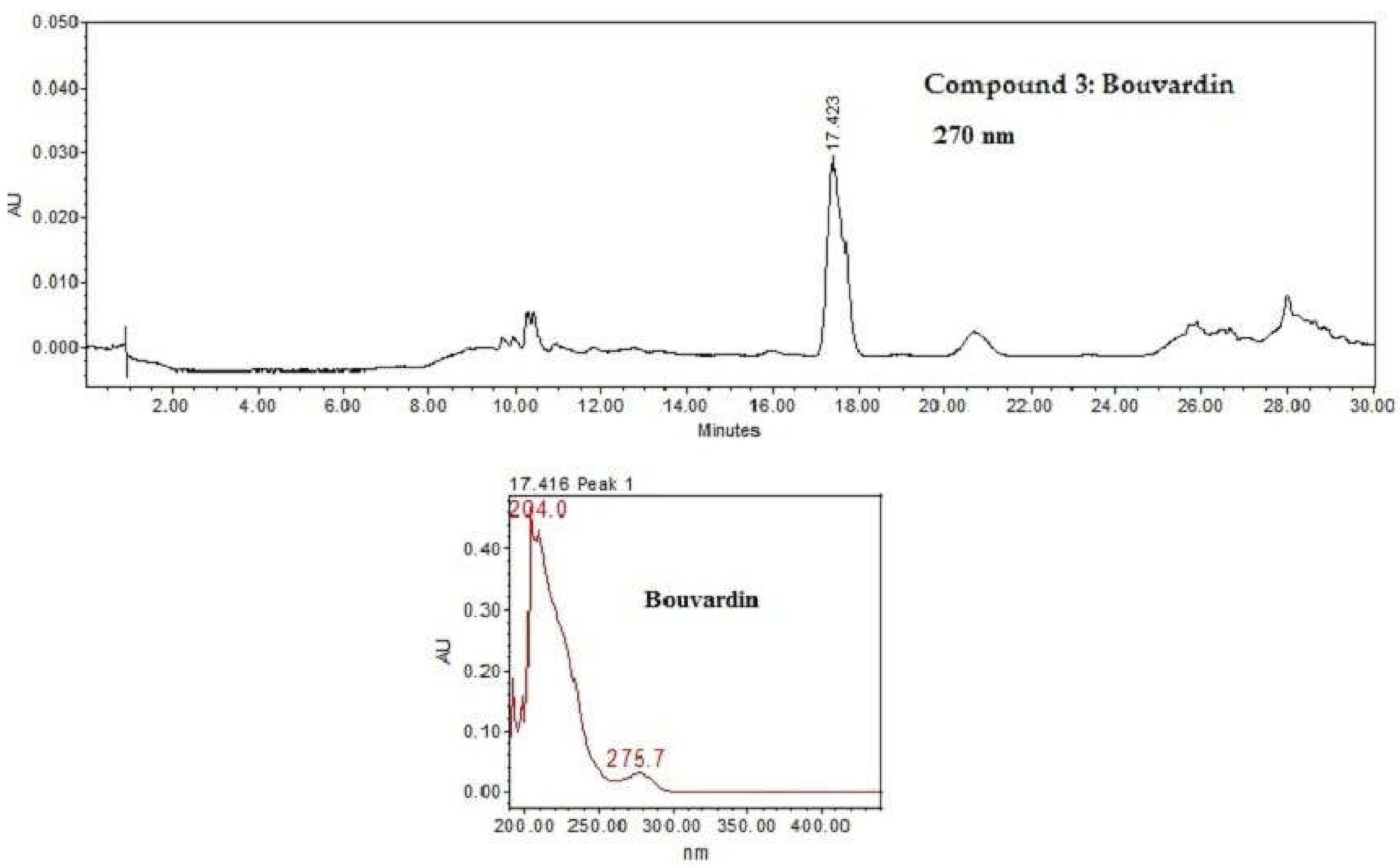

Figure S18. Spectrum UV of bouvardin (**3**)

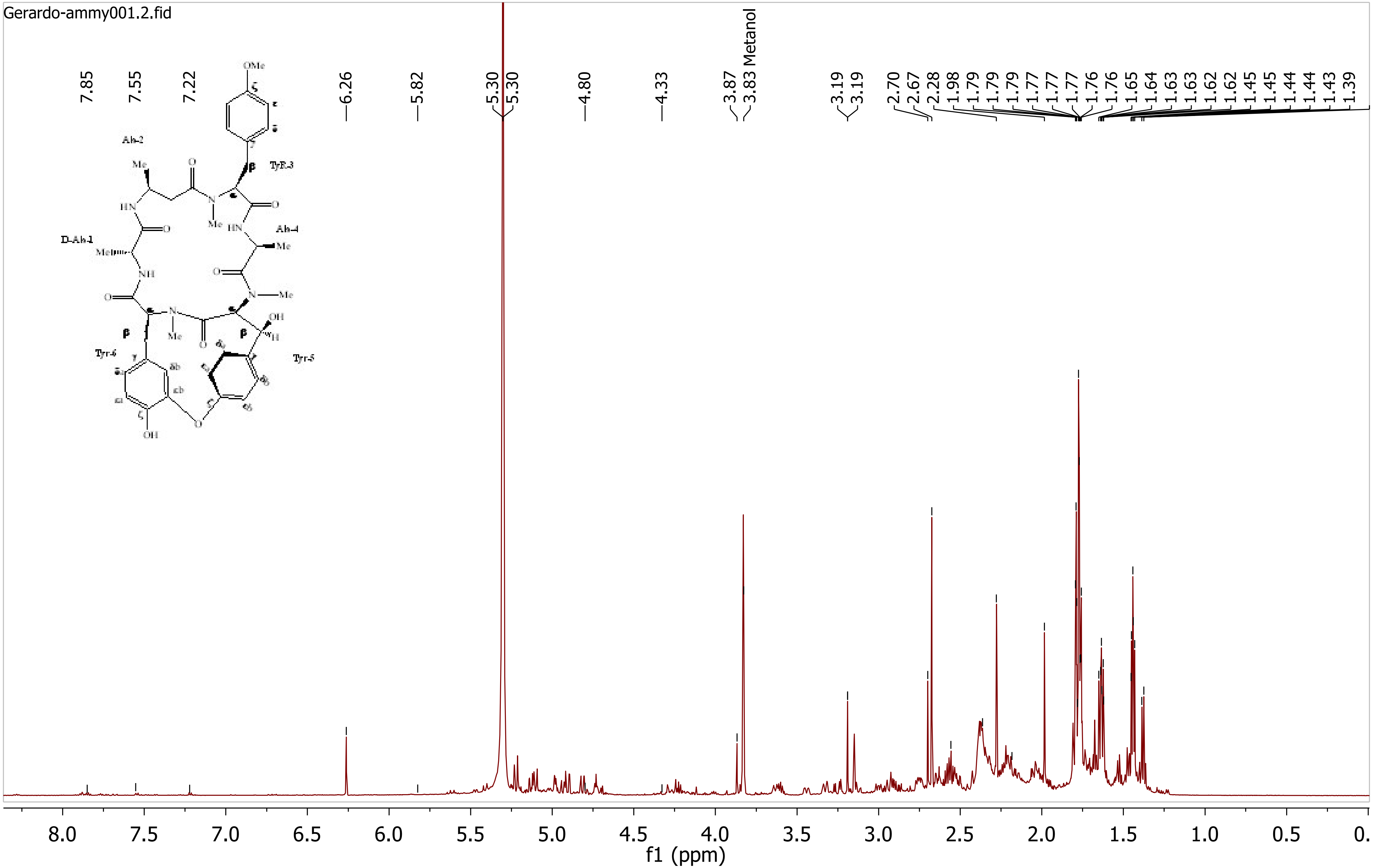

Figure S19. ^{1}H NMR (CD_3OD, 400 MHz) of bouvardin (**3**)

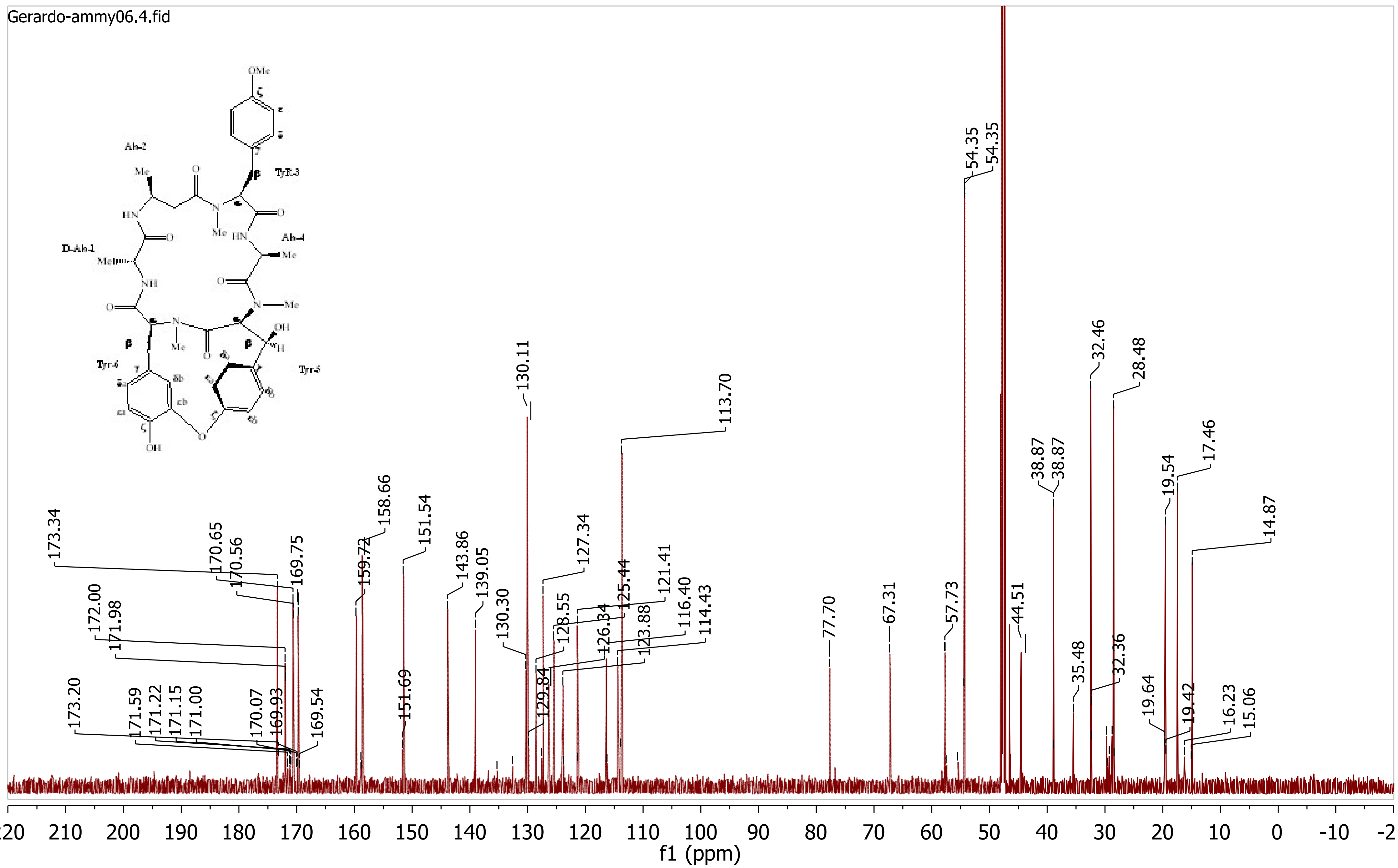

Figure S20. ^{13}CNMR (CD_3OD, 100 MHz) of bouvardin (**3**)

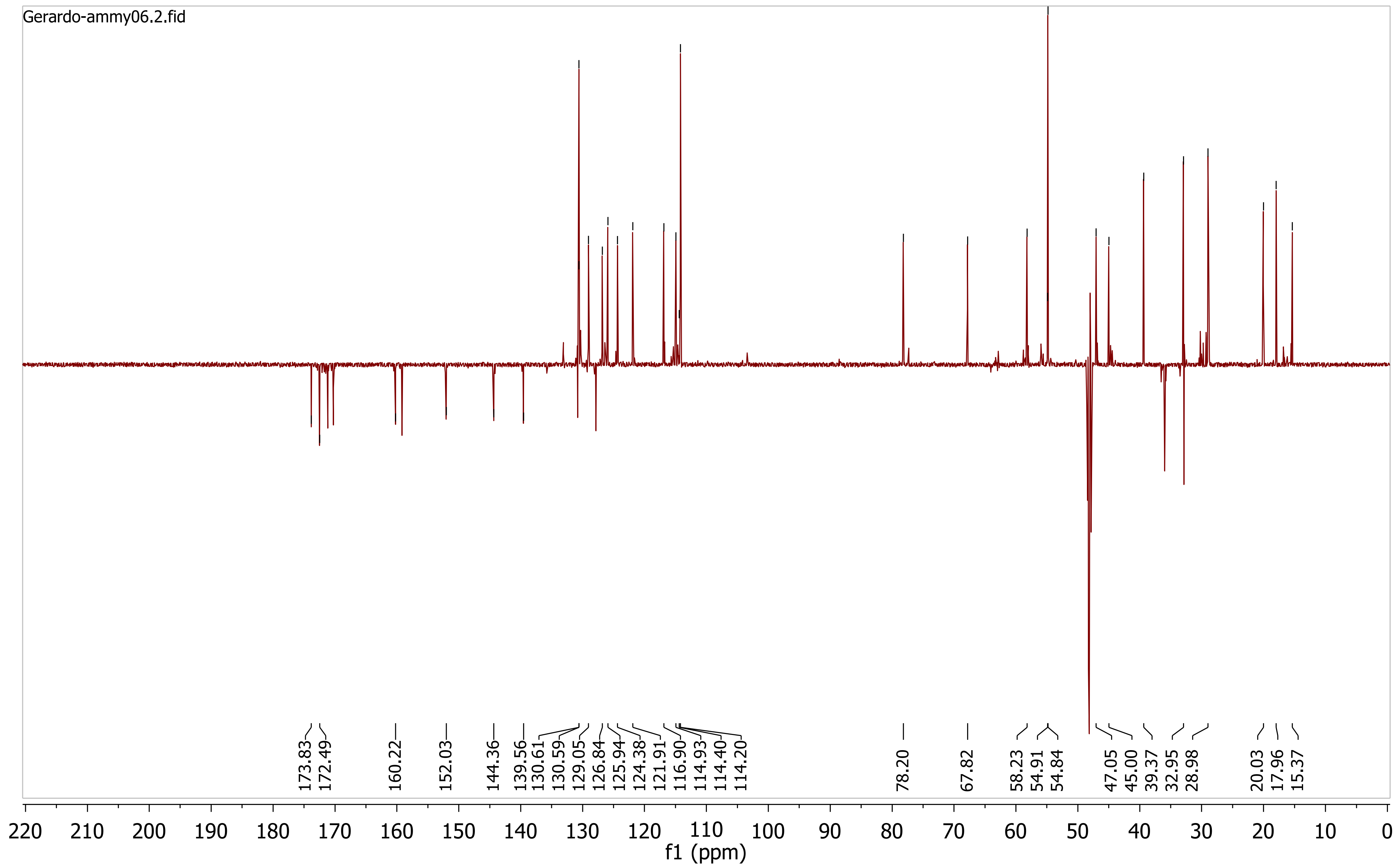

Figure S21. ^{13}C (DEPT) NMR (CD_3OD, 100 MHz) of bouvardin (**3**)

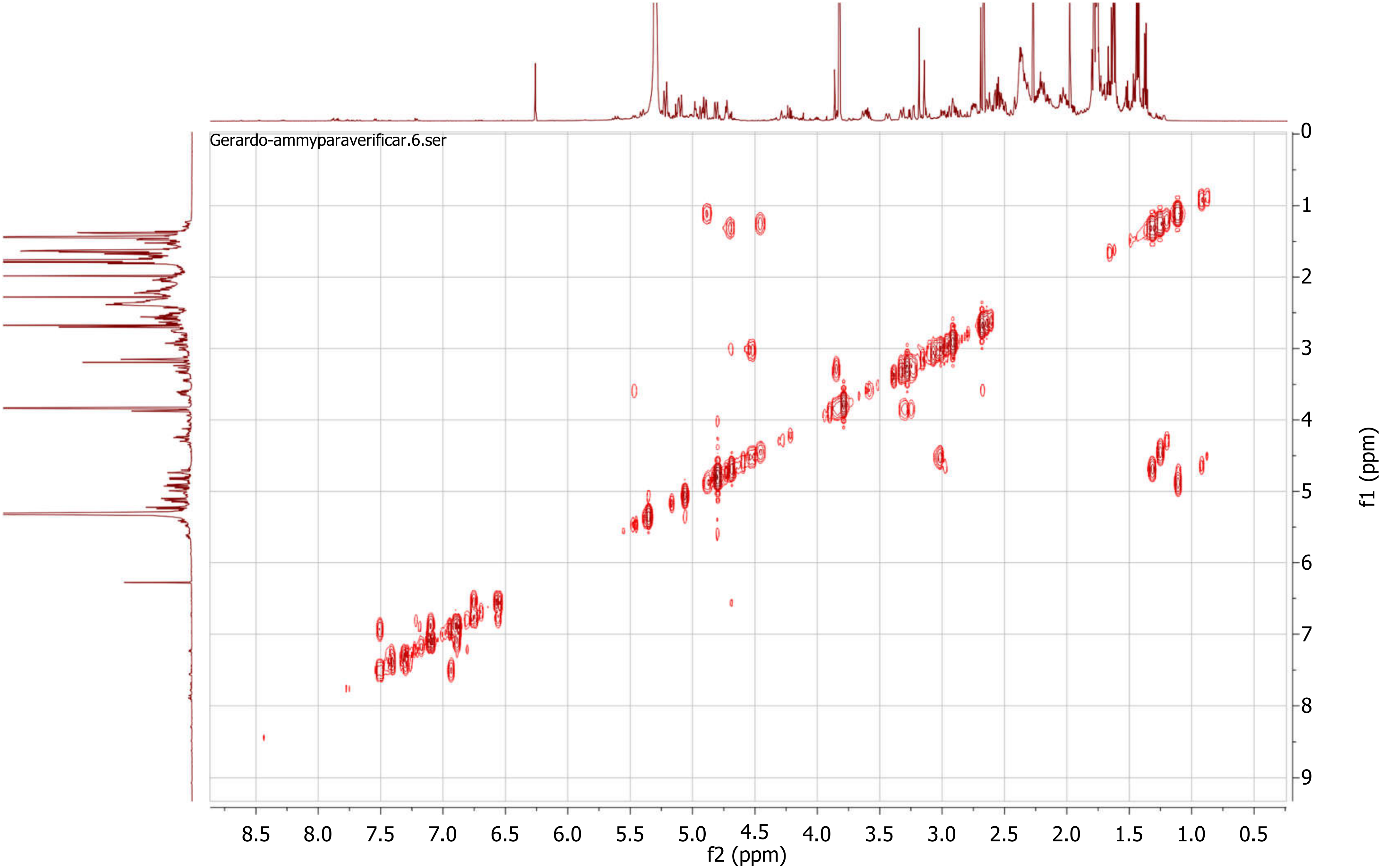

Figure S22. ^1H-^1H COSY NMR (CD_3OD, 400 MHz) of bouvardin (**3**)

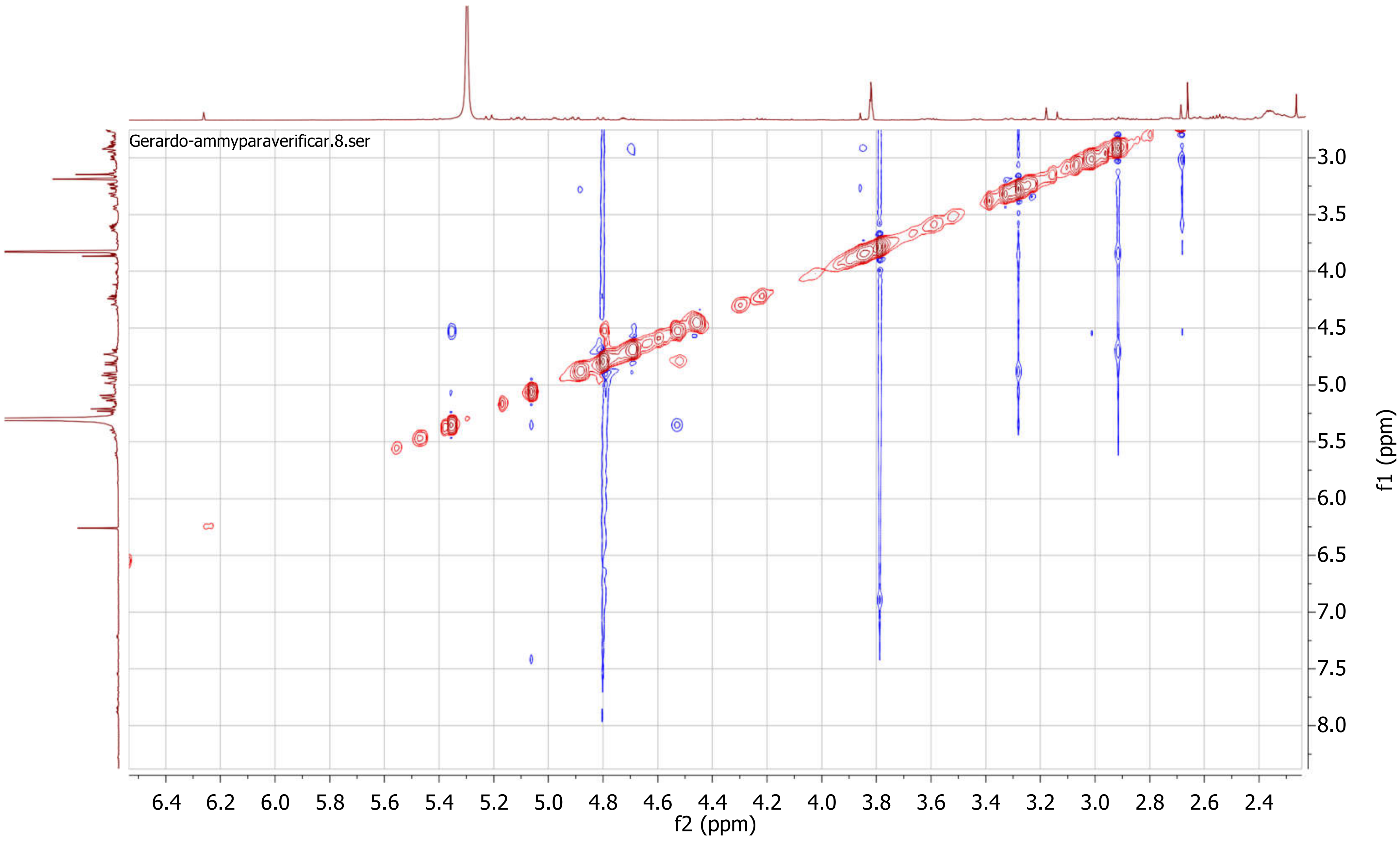

Figure S23. ^{1}H-^{1}H NOESY NMR (CD_3OD, 400 MHz) of bouvardin (**3**)

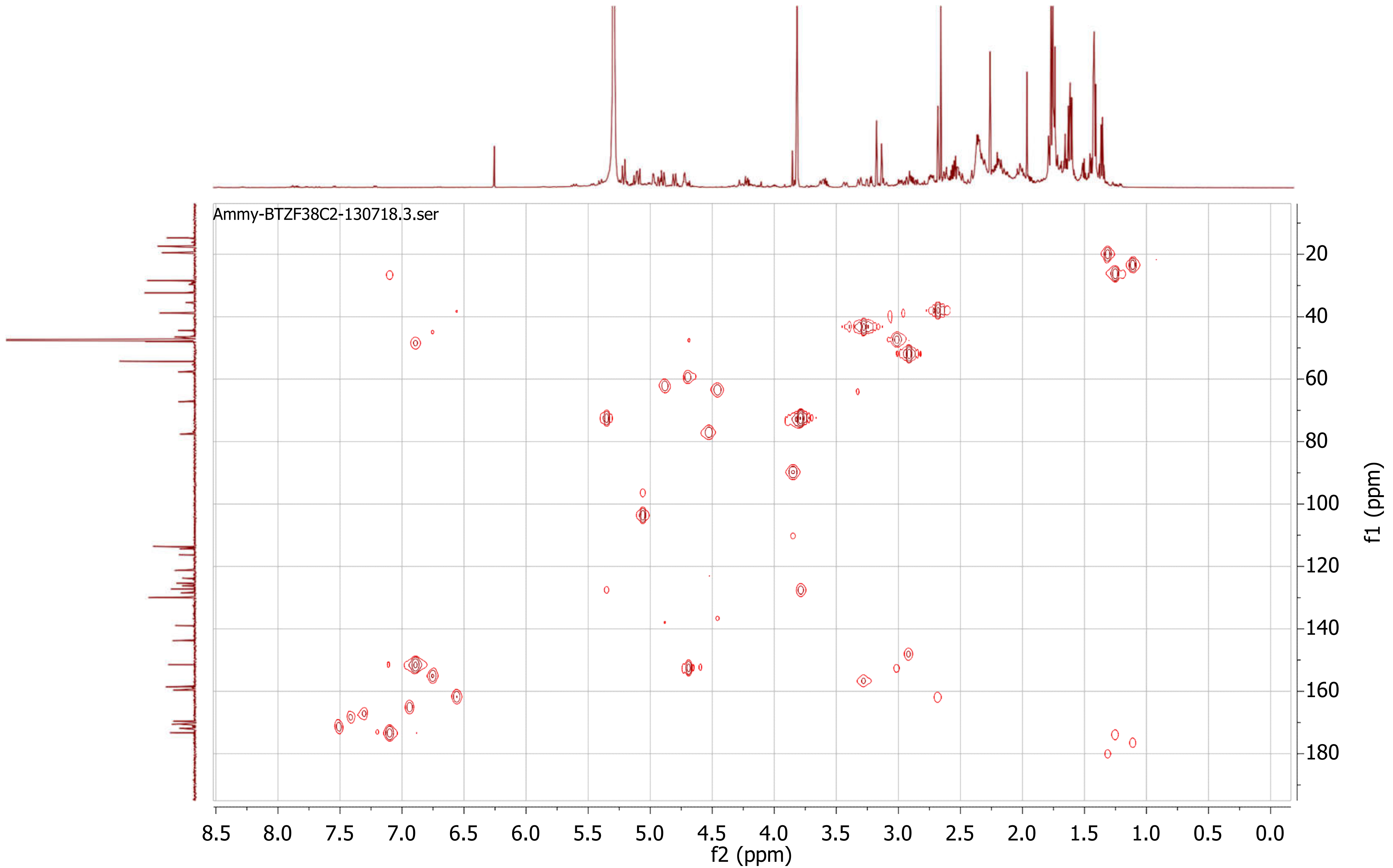

Figure S24. ^{1}H-^{13}C (HMBC) NMR (CD_3OD), 400 MHz) of bouvardin (**3**)

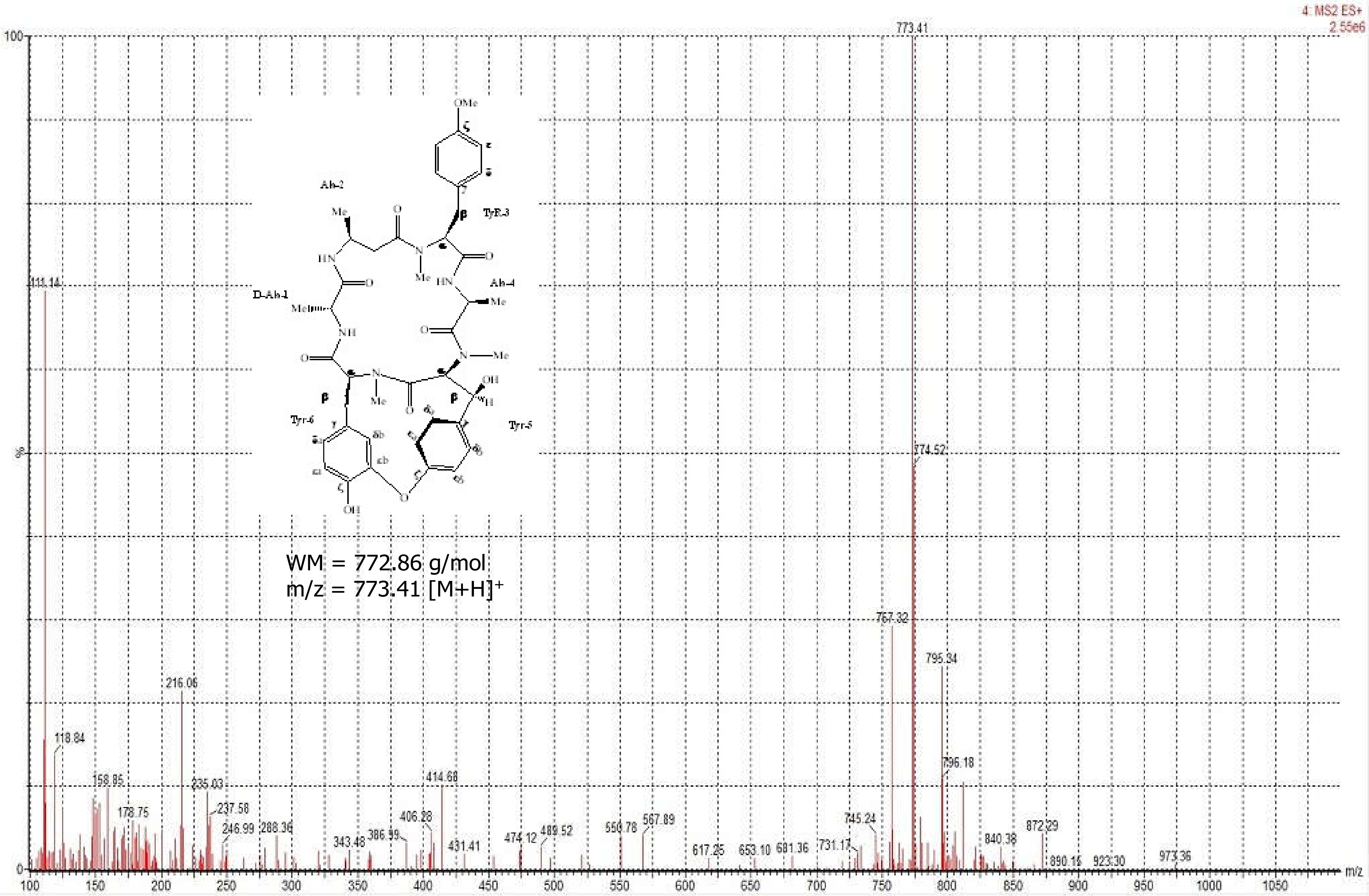

Figure S25. Mass spectrum (MS) of bouvardin (**3**)

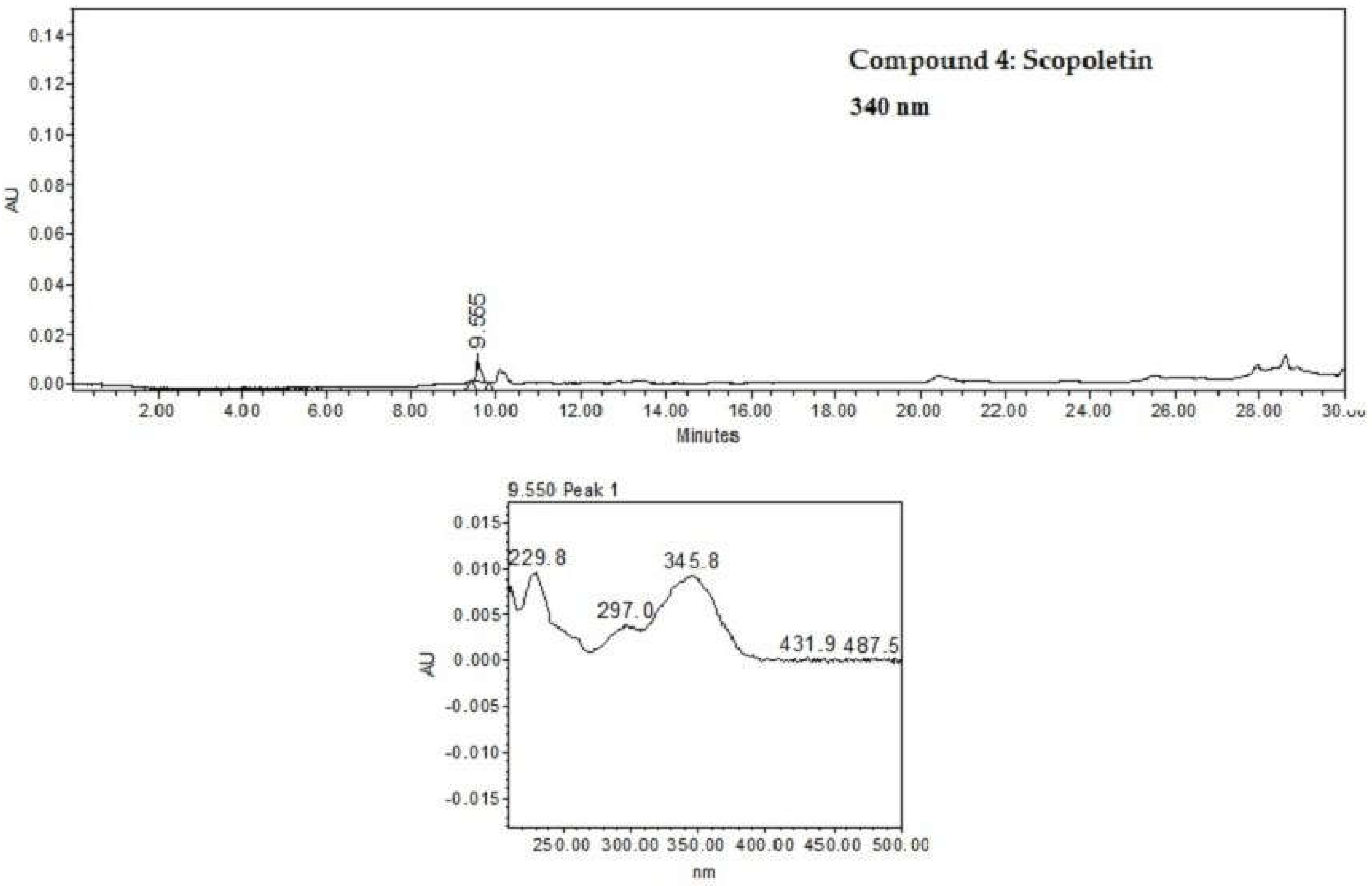

Fig S26. Spectrum UV of Scopoletin (**4**)

Gerardo-nmr-18.1.1.1r
Ammy-McNdC24-16-220517-PROTON

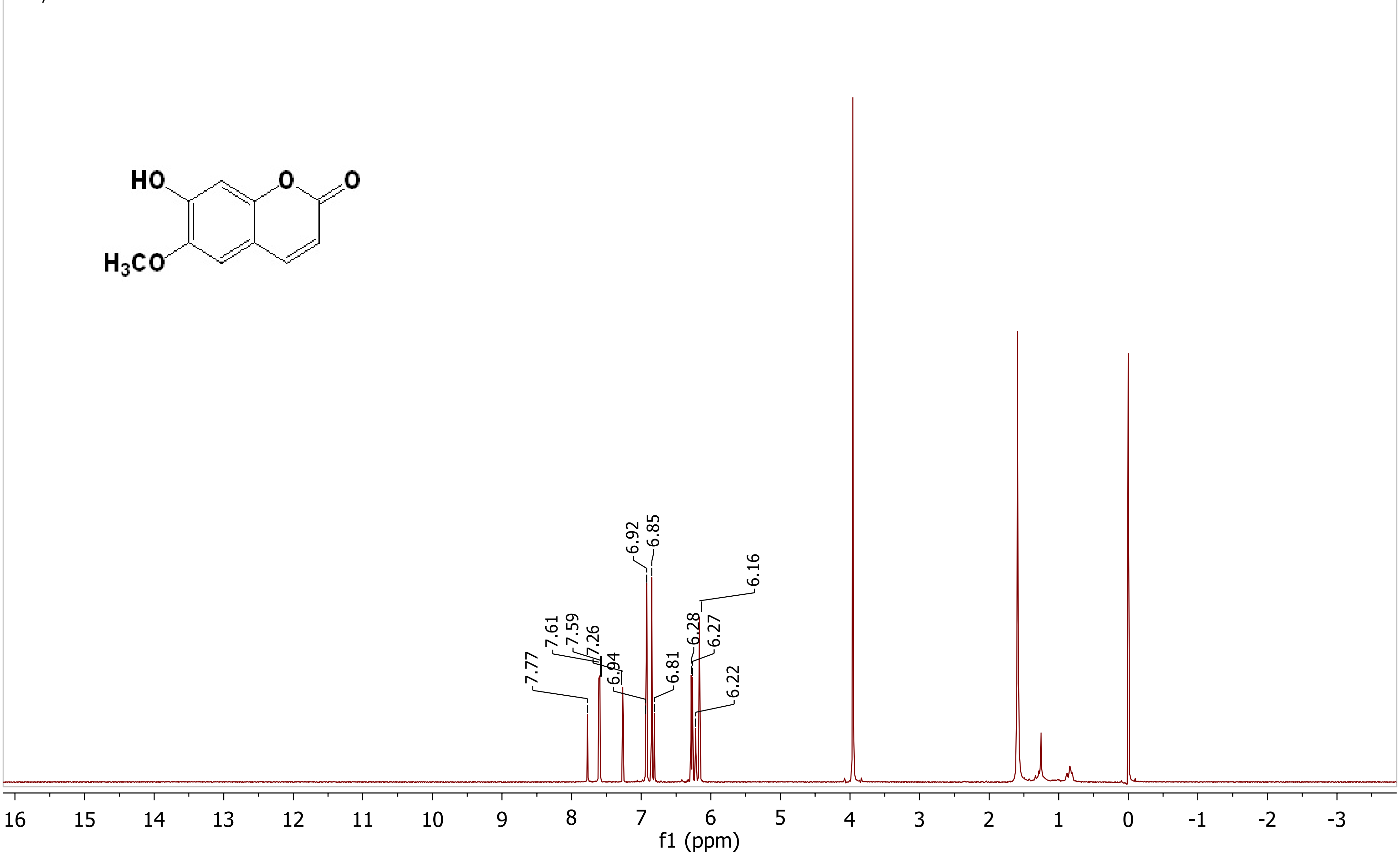

Figure S27. ^{1}H NMR (CD_3O, 600 MHz) of scopoletin (**4**)

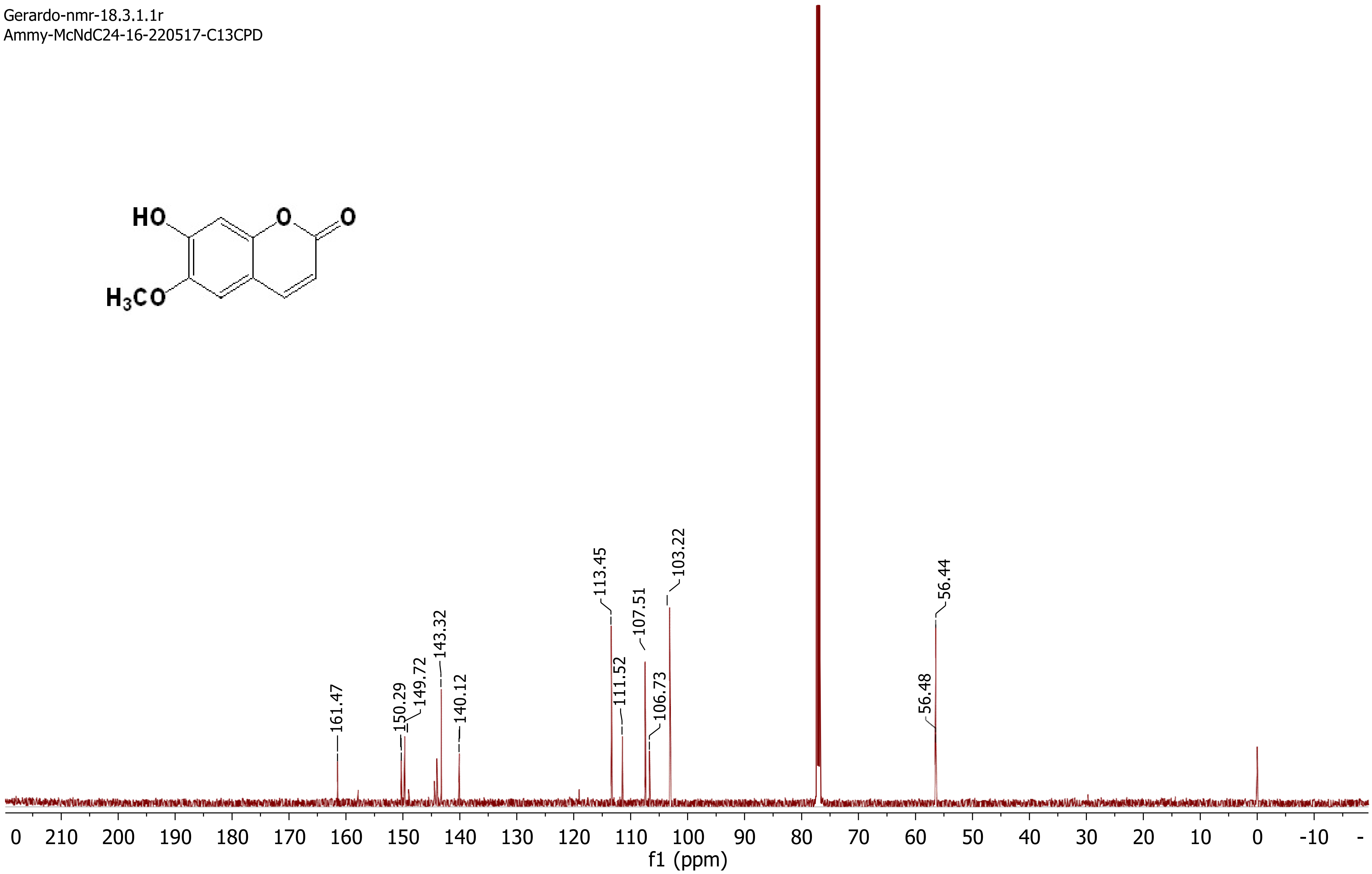

Figura S28. ^{13}C NMR (CD_3OD, 150 MHz) of scopoletin (**4**)

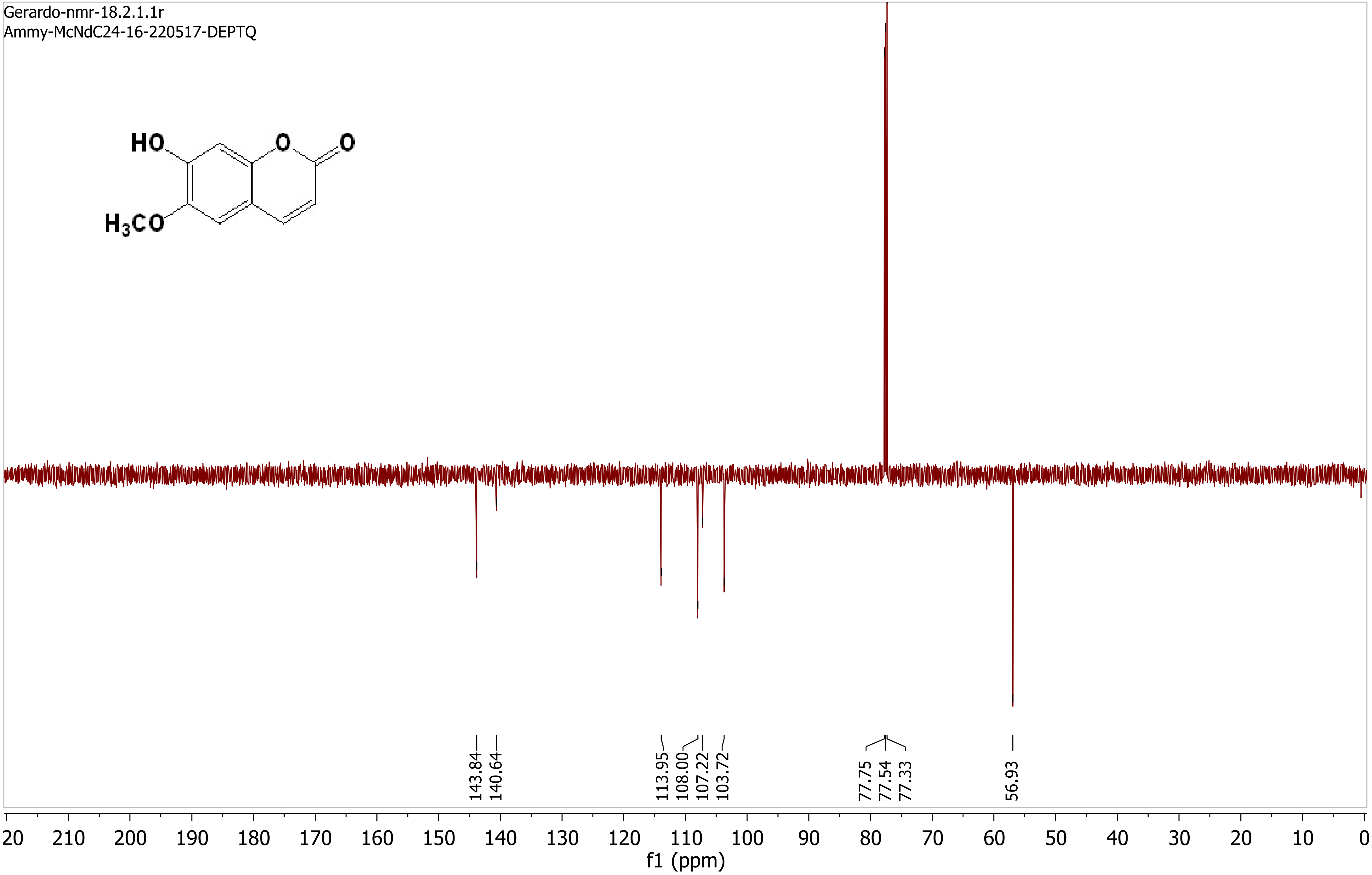

Figure S29. ^{13}C (DEPT) NMR (CD_3OD, 150 MHz) of scopoletin (**4**)

Table S1: ^{13}C NMR (CD_3OD, 100 MHz) of bouvardin (**3**), ^1H-^{13}C (HSQC) NMR (CD_3OD, 400 MHz) and ^{13}C NMR ($CDCL_3$, Bates *et al*., 1983)

	Experimental ^{13}C NMR of Bouvardin (3)	^1H-^{13}C (HSQC) NMR of Bouvardin (3)	Referencia ^{13}C NMR Bouvardin (Bates *et al.,* 1983)
**Ala-2β**	16.39	1.29 (d *J*=7.03)	16.4q
**Ala-4β**	18.93	1.08 (d *J*=6.64)	18.5q
**Ala-1β**	21.09	1.22 (d *J*=6.64)	20.5q
**Tyr-6 N-Me**	30.01	2.67 (s)	29.2q
**Tyr-5 N-Me**	33.80	3.25 (s)	33.0q
**Tyr-3β**	33.98		32.7t
**Tyr-6β**	36.5	2.99 (s)	35.9t
**Tyr-5β**	79.11	5.03 (s)	78.5 d
**Tyr-3 N-Me**	40.38	2.86 (s)	39.7q
**Ala- 1 α**	45.96	4.67 (s)	44.9d
**Ala-4α**	46.18		46.5d
**Ala-2α**	48.1	4.87 (s)	47.9d
**Tyr-5α**	55.74	5.32 (d *J*=1.95)	53.9d
**Tyr-3 0-Me**	55.81	3.76 (s)	55.3d
**Tyr-6α**	59.11	4.45 (s)	57.8d
**Tyr-3α**	68.74	3.82 (s)	68.75d

**Tyr-6δ$_a$**	115.13	4.65 (s)	113.0d
**Tyr-3£**	116.05	6.86 (d *J*=8.60)	114.1d
**Tyr-6 £$_a$**	117.87	6.73 (s)	116.1d
**Tyr-6δb**	122.89	6.53 (d *J*=1.56)	124.2d
**Tyr-5£a**	125.37	6.90(dd *J*=8.60; 10.55)	125.7d
**Tyr-6 ϒ**	126.95	7.27(dd *J*= 8.60; 10.55)	127.2s
**Tyr-3δ**	131.50	7.07(d *J*= 8.60)	130.3d
**Tyr-3ϒ**	131.71	7.48 (dd *J*= 8.60; 10.55)	130.7s
**Tyr-5 δ$_a$**	127.17		126.9d
**Tyr-5 δ$_b$**	128.78		128.5d
**Tyr-5ϒ**	140.54		139.8s
**Tyr-6ζ**	145.28		143.2s
**Tyr-6£$_b$**	152.96		151.0s
**Tyr-5ζ**	160.06		158.9s
**Tyr-3ζ**	161.10		158.4s
**Tyr-6 CO**	167.81		168.3s
**Tyr-5 CO**	171.25		170.0s
**Tyr-3 CO**	171.98		170.8s
**Ala-4 CO**	172.10		172.2s
**Ala-1 CO**	173.39		172.2s
**Ala-2 CO**	174.78		172.5s

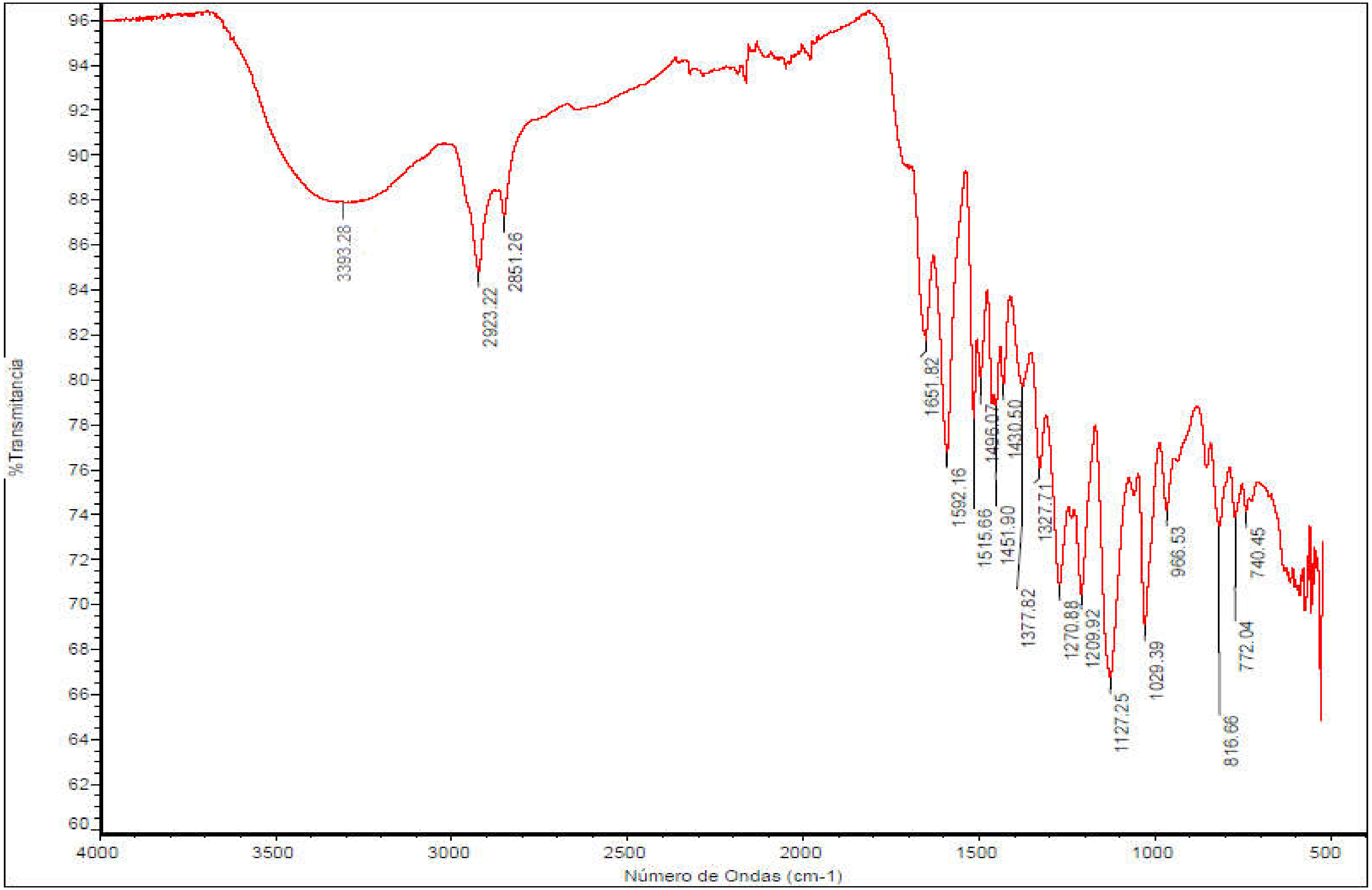

Figure S30. IF spectroscopy ternifoliol (**1**)

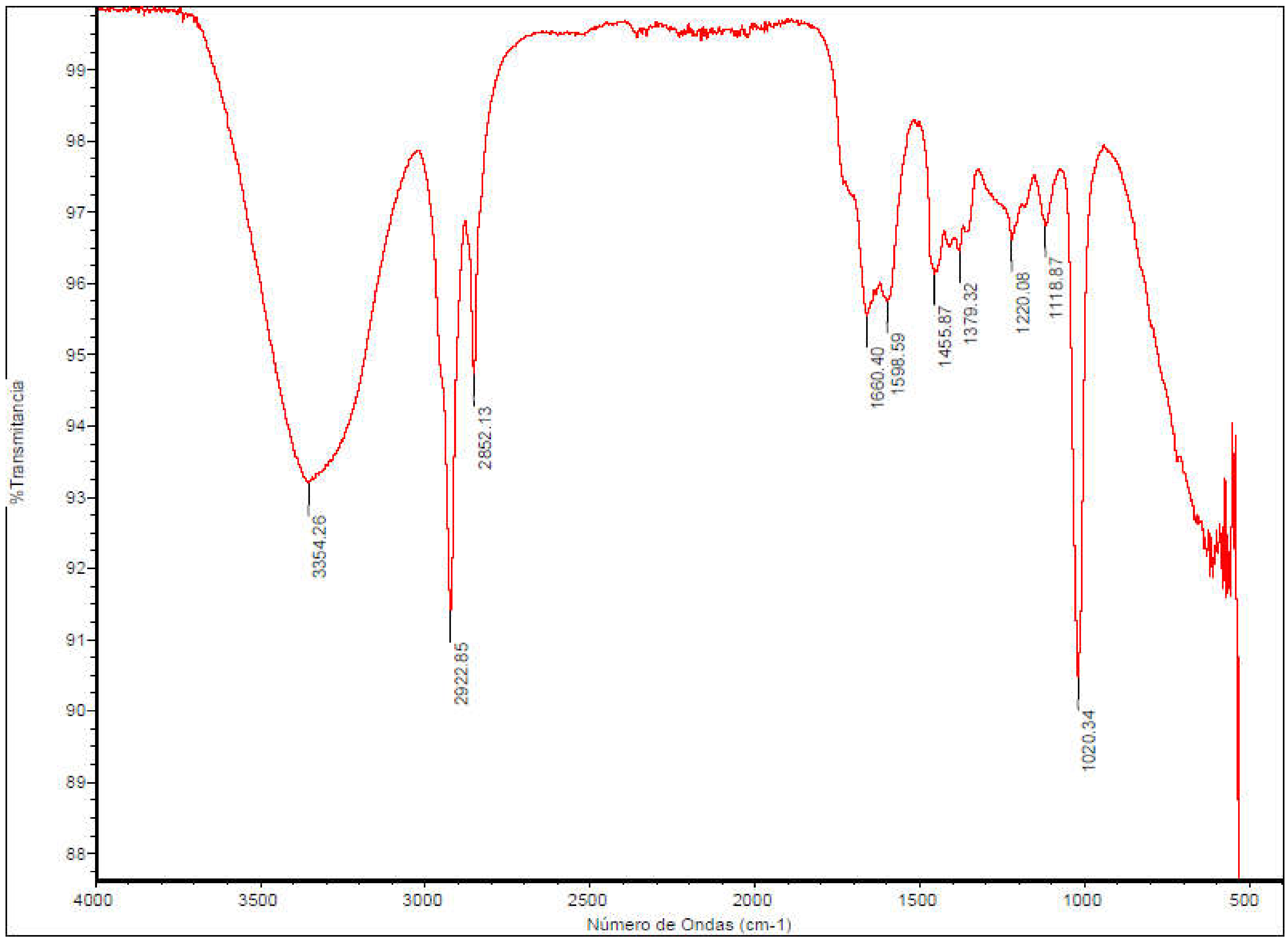

Figure S31. IF spectroscopy ternifolial (2)
